# Improved DC Characteristic Modeling and Small-Signal Investigation of GaN Schottky Barrier Diodes

**DOI:** 10.3390/mi17091078

**Published:** 2026-09-12

**Authors:** Chuangye Wang, Liyue Tang, Ao Zhang, Jiali Cheng, Caoyu Li, Shuman Mao, Yuehang Xu, Qi He, Tao Guo, Kai Wang, Chang Wu

**Affiliations:** 1 School of Electronic Engineering, Jiangsu Ocean University, Lianyungang 222005, China; 2024210612@jou.edu.cn (C.W.); 2024121357@jou.edu.cn (L.T.); 2 School of Microelectronics, Nantong University, Nantong 226019, China; 3 Zhejiang Key Laboratory of Information and Energy Integrated Microsystem, Yangtze Delta Region Institute (Huzhou), University of Electronic Science and Technology of China, Huzhou 313001, China; licaoyu@csj.uestc.edu.cn (C.L.); maoshuman@csj.uestc.edu.cn (S.M.); yuehangxu@uestc.edu.cn (Y.X.); 4 Hubei Jiufengshan Laboratory, Wuhan 430074, China; heqi@jfslab.com.cn (Q.H.); guotao@jfslab.com.cn (T.G.); wangkai1@jfslab.com.cn (K.W.); wuchang@jfslab.com.cn (C.W.)

**Keywords:** GaN SBD, parameter extraction, small-signal modeling, DC characteristics

## Abstract

This paper presents a complete modeling flow for GaN Schottky barrier diodes (SBDs), encompassing parasitic parameter de-embedding, small-signal equivalent circuit extraction, and DC characteristic analysis. The open/short de-embedding method is adopted to extract the parasitic parameters of the Ground–Signal–Ground (GSG) pads. A bias-partitioning strategy is employed to extract the intrinsic small-signal parameters, and the depletion capacitance model is used to physically fit the C-V characteristics. The forward Direct Current (DC) conduction current is described by the thermionic emission model, while the reverse leakage is modeled using a piecewise approach: the Poole–Frenkel (PF) trap-assisted emission model is applied in the low-bias region, and a double-exponential decay empirical model is introduced in the high-bias region, achieving high-precision fitting over the full bias range (−100 V~3 V). More importantly, this paper identifies a significant discrepancy between the series resistance extracted from DC measurements and that from Radio Frequency (RF) measurements, and attributes it to the frequency dispersion effect induced by trap states. The study demonstrates that combining DC and high-frequency characterization not only enables the construction of an accurate modeling framework, but also reveals the trap-related physical mechanisms within the device.

## 1. Introduction

As a representative third-generation wide-bandgap semiconductor, GaN offers outstanding physical properties—including a large bandgap, a high breakdown electric field, and high electron mobility—making it highly advantageous for high-temperature, high-frequency, and high-power electronic devices. GaN SBDs, characterized by fast switching speed, low on-resistance, and high cut-off frequency, hold significant potential for applications such as microwave rectification, frequency multiplication, and power switching [1]. Furthermore, through structural optimization approaches such as recessed anodes and low-work-function metals, the forward turn-on and reverse leakage characteristics of GaN SBDs have been substantially improved [2].

In the high-frequency modeling of GaN SBDs, accurate parasitic parameter extraction and the establishment of a small-signal equivalent circuit are critical prerequisites for circuit design. Koolen et al. [3] proposed an improved on-wafer high-frequency de-embedding technique, which has since become one of the standard methods for parasitic parameter removal. Zhang et al. [4] established an equivalent circuit model for millimeter-wave Schottky diodes and validated it through on-wafer S-parameter measurements. With regard to on-wafer measurement techniques, Zhang and Gao [5] analyzed both single-port and two-port measurement methods for Schottky diodes at millimeter-wave frequencies, designed corresponding test layouts, and provided small-signal and large-signal equivalent circuit models. Huang et al. [6] proposed a parameter extraction method for the millimeter-wave equivalent circuit model of Schottky diodes. Tang et al. [7] developed a method for systematically extracting high-frequency model parameters of Schottky diodes from broadband S-parameters; this approach enables direct calculation of equivalent circuit elements without numerical optimization, and notably resolves difficulties in extracting inductance and resistance parameters, with validation up to 110 GHz and 600 GHz under CPW and suspended coplanar waveguide configurations. More recently, Orfao et al. [8] established a small-dimension diode model for extracting Schottky parameters from S-parameter measurements, targeting GaN-on-sapphire SBDs for frequency multiplier applications, and the obtained capacitance parameters agreed well with C–V measurement results from large-dimension diodes. However, the aforementioned studies focus primarily on the extraction methodology itself, with limited attention paid to the physical significance of the extracted parameters, particularly the correlation between DC and RF extracted values, which may reveal trap-related dispersion effects in GaN materials.

For DC modeling, researchers fabricated vertical SBDs on homoepitaxial GaN and proposed a reverse leakage model based on electron tunneling through dislocation-related continuous defect states, indicating that electric-field-assisted transport of electrons over a locally lowered Schottky barrier via dislocation states is a significant source of reverse leakage [9]. At the device design level, studies have focused on field-plate structure optimization, proposing that reducing the field-plate pinch-off voltage can effectively suppress the reverse leakage current [10]. Furthermore, a composite model incorporating multiple leakage mechanisms has been established, and simulations have analyzed the dominant roles of PF emission, variable-range hopping, and Fowler–Nordheim tunneling in different bias intervals [11], providing an important basis for the piecewise modeling of reverse leakage in this work.

This paper presents a systematic study of GaN SBDs, covering parasitic parameter extraction, small-signal modeling, and DC characteristic analysis. Unlike prior work, the main contributions of this paper are twofold: (1) a complete modeling flow from de-embedding to device characteristic fitting is established, achieving high-precision fitting over the full small-signal (0.1~50 GHz) and DC (−100 V~3 V) ranges; (2) the series resistance values extracted from DC and RF measurements are compared, revealing a significant discrepancy (3.3 Ω vs. 5.6 Ω), which is attributed to the frequency dispersion effect induced by trap states in the GaN epitaxial layer, thereby providing a basis for the non-destructive evaluation of device material quality. The remainder of this paper is organized as follows: Section 2 details the equivalent circuit models of the open/short de-embedding test structures and the parasitic parameter extraction method; Section 3 establishes the small-signal equivalent circuit model of the device, completing the de-embedding process and the extraction and verification of intrinsic parameters across different bias regions; Section 4 conducts piecewise modeling of the forward conduction and reverse leakage characteristics, adopting the PF trap-assisted emission model in the low-bias region and introducing an empirical model that captures the current saturation trend in the high-bias region, thereby achieving high-precision fitting over the full bias range; Section 5 systematically discusses the consistency between the fitted results of each modeling step and the measured data, validating the effectiveness of the integrated modeling flow; and Section 6 concludes the paper.

## 2. Test Structures and Parasitic Parameter Extraction

### 2.1. Introduction of the GaN SBD Devices

**Figure 1:**
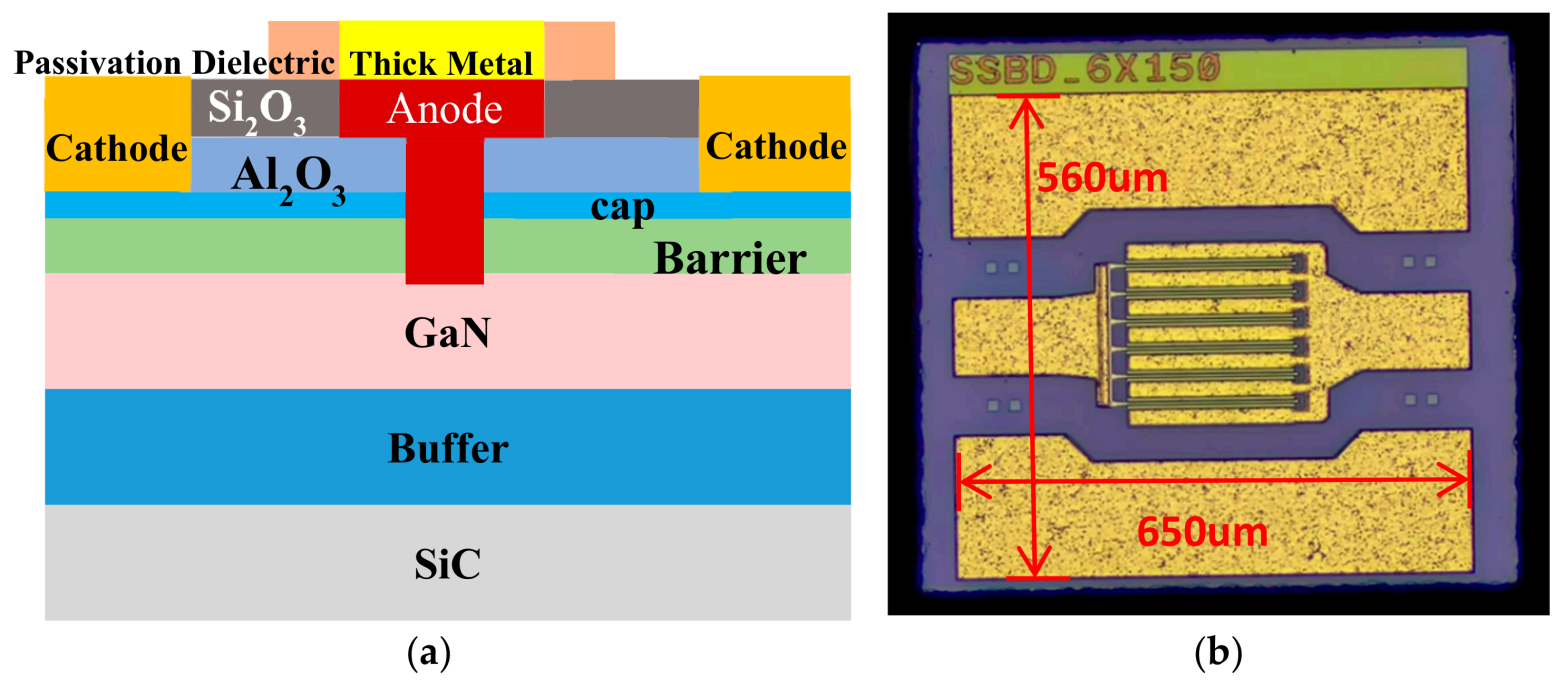
Device structure: (**a**) schematic cross-section of the device; (**b**) device test structure layout.

The GaN SBD studied in this work was fabricated using a 0.25 μm process developed at Jiufengshan Laboratory. The device, SSBD_6×150, features an interdigitated structure which comprises six parallel anode fingers, each with a length of 150 μm. The GaN SBD structure is grown on a SiC substrate, as shown in Figure 1a, consisting of a GaN cap, an AlGaN barrier, a GaN channel layer and an undoped GaN buffer. Figure 1b presents the test structure layout of the device.

**Figure 2:**
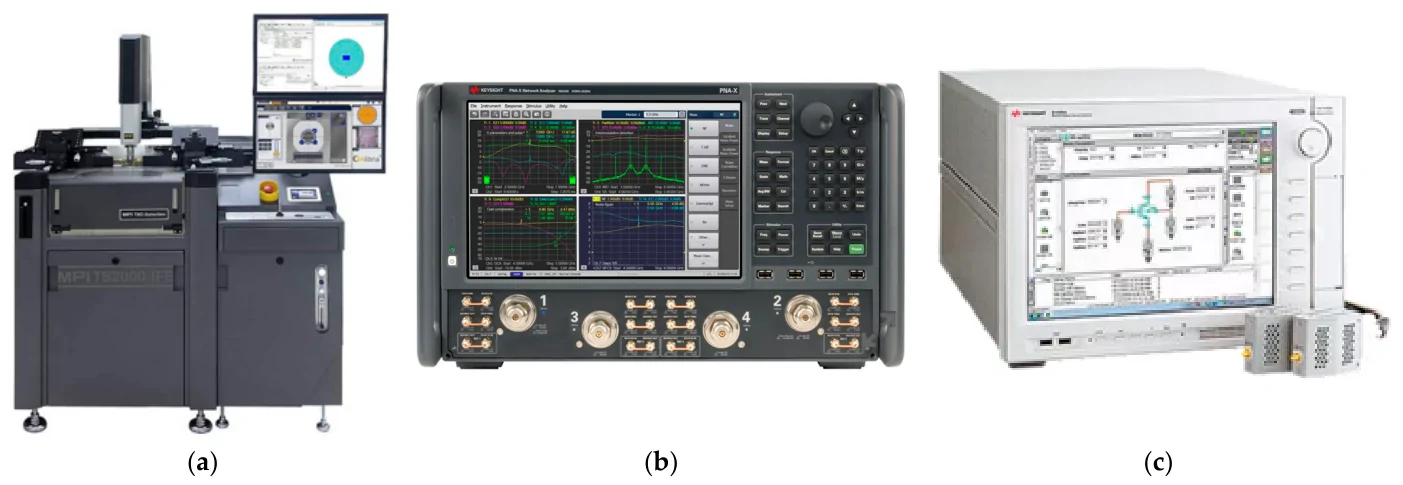
Test system: (**a**) MPI TS2000-IFE, (**b**) Keysight N5245B, and (**c**) Keysight B1500A.

DC I-V relations and RF and microwave multi-bias S-parameters from 0.1 to 50 GHz are measured with a measurement system, which consists of an MPI probe station TS2000-IFE, a Keysight vector network analyzer N5245B and a Keysight semiconductor parameter analyzer B1500A. The test system is shown in Figure 2. (Images courtesy of MPI Corporation, Zhubei City, Taiwan and Keysight Technologies, Santa Rosa, CA, USA).

### 2.2. Test Structures and Equivalent Circuit Models

In the high-frequency modeling of GaN SBD devices, accurate extraction of parasitic parameters is a critical prerequisite, as the accuracy of de-embedding directly affects the fitting precision of the small-signal model. Commonly used de-embedding methods include the open de-embedding method, the open–short de-embedding method, the L-2L method, and the TRL method, among others. In this work, the open–short de-embedding method is adopted, and the corresponding open and short equivalent circuit models are established based on the GSG pad configuration. This section describes in detail the open and short test structures used for parasitic parameter extraction, as well as the complete de-embedding and parameter extraction procedure [12,13].

**Figure 3:**
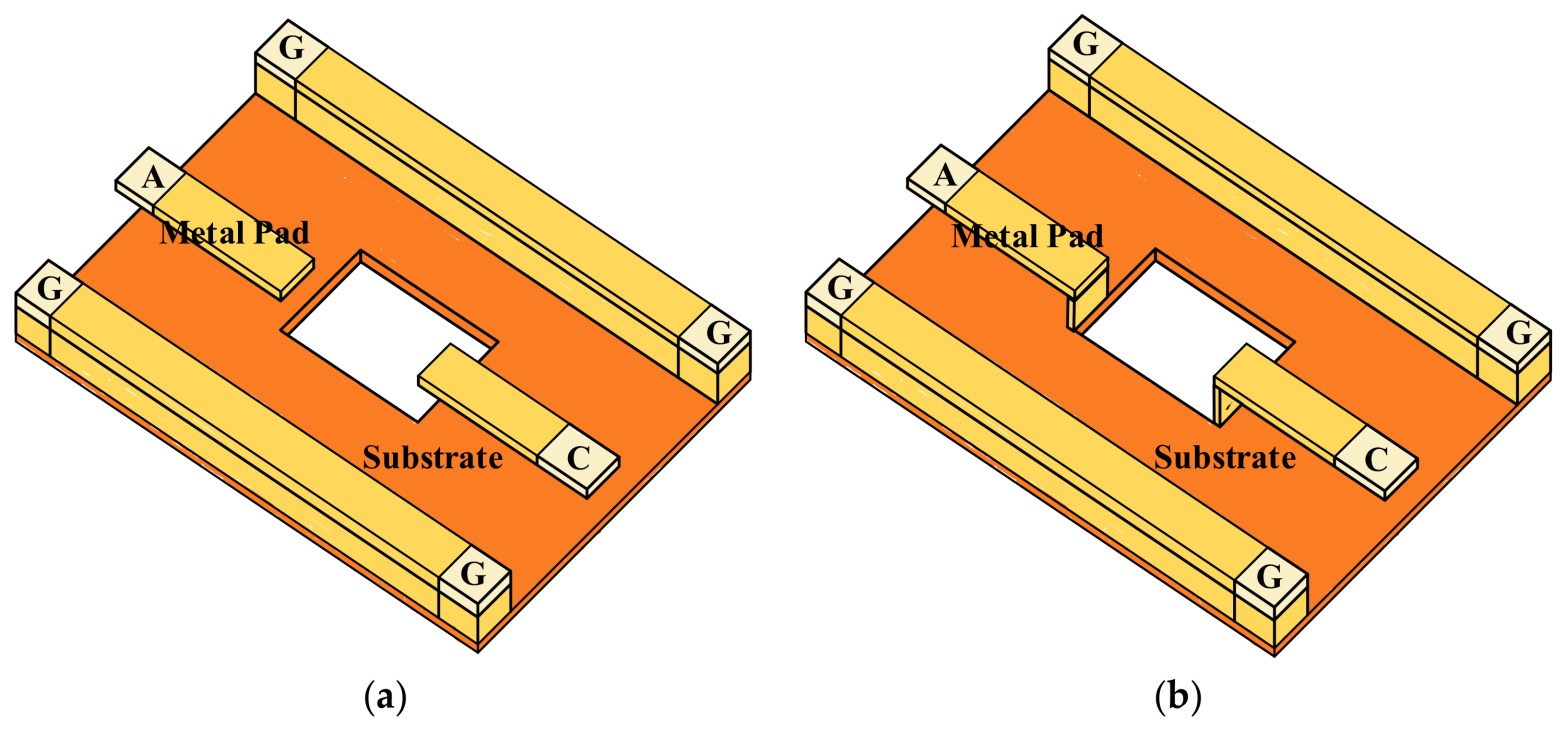
Test structure: (**a**) open test structure; (**b**) short test structure.

The open–short de-embedding method assumes that all parallel parasitics are located at the pads, while all series parasitics are lumped onto the interconnects. This method requires an open test structure and a short test structure. Figure 3 shows schematic diagrams of the test structures, in which Figure 3a depicts the open test structure, which contains neither the device under test nor the interconnects, and Figure 3b shows the short test structure, in which the input and output ports are shorted to the substrate [12,13].

**Figure 4:**
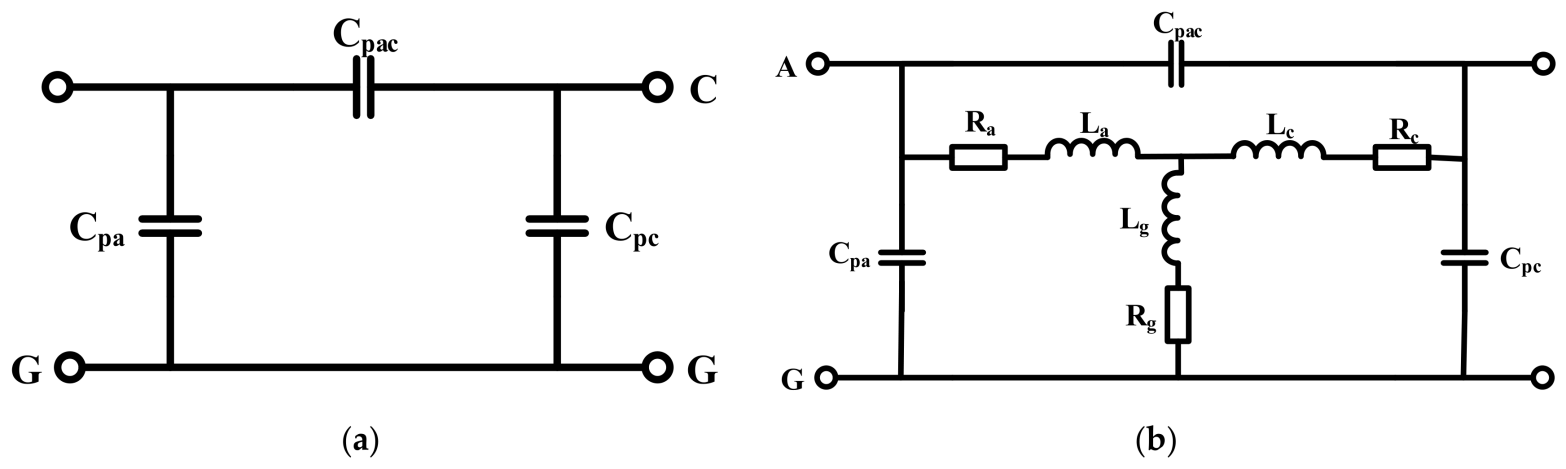
Equivalent circuit models of the test structures: (**a**) equivalent circuit model of the open test structure; (**b**) equivalent circuit model of the short test structure.

In the de-embedding process, the equivalent circuit models corresponding to the test structures must first be established. Figure 4 illustrates the equivalent circuit models of the test structures, in which Figure 4a shows the equivalent circuit model of the open test structure and Figure 4b shows that of the short test structure.

### 2.3. Parasitic Parameter Extraction

*C_pa_* represents the capacitance between the signal input pad and the substrate, *C_pc_* represents the capacitance between the signal output pad and the substrate, and *C_pac_* represents the coupling capacitance between the signal input and signal output pads. *L_a_*, *L_c_*, and *L_g_* denote the parasitic inductances of the signal input, signal output, and substrate interconnects, respectively. *R_a_*, *R_c_*, and *R_g_* denote the parasitic resistances of the signal input, signal output, and substrate interconnects, respectively.

For the open test structure, its Y-parameter matrix *Y_open_* is given by [12,13,14,15]:

(1) $$Y_{open}=\left[\begin{array}{cc} j\omega (C_{pac}+C_{pa}) & -j\omega C_{pac}\\ -j\omega C_{pac} & j\omega (C_{pac}+C_{pc}) \end{array}\right]$$

**Figure 5:**
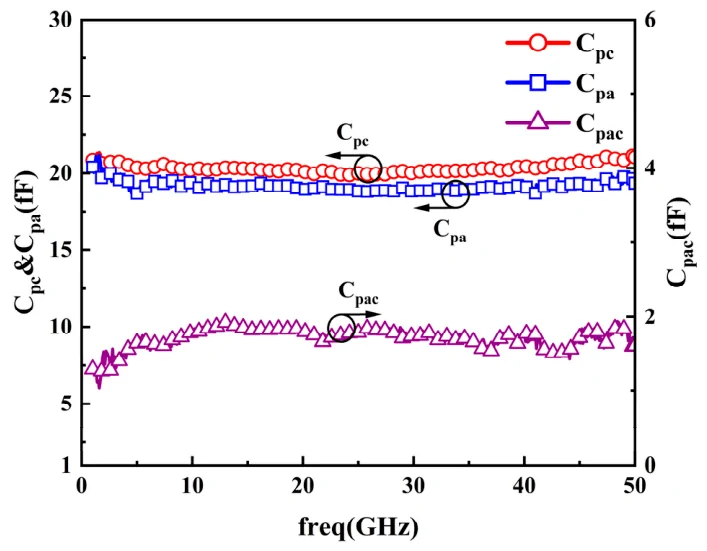
Parasitic capacitances of the open test structure.

From the Y-parameter matrix, the expressions for the parasitic parameters can be obtained. Using the measured S-parameters of the device as input, the parameter extraction was performed in ADS by implementing the self-built device equivalent circuit, and the extraction results are shown in Figure 5.

For the short test structure, the effects of the external parasitic pads must be eliminated before extracting the feedline parasitics. The calculation formula is as follows [12,13,14,15]:

(2) $$Y_{short1}=Y_{short}-Y_{open},$$

where *Y_short_* is the admittance Y-parameter of the short test structure, *Y_short_*_1_ represents the Y-parameter matrix with the pad parasitics removed, and converting *Y_short_*_1_ to *Z_short_*_1_ gives [12,13,14,15]:

(3) $$Z_{short1}=\left[\begin{array}{cc} R_{a}+R_{g}+j\omega (L_{a}+L_{g}) & R_{g}+j\omega L_{g}\\ R_{g}+j\omega L_{g} & R_{c}+R_{g}+j\omega (L_{c}+L_{g}) \end{array}\right]$$

**Figure 6:**
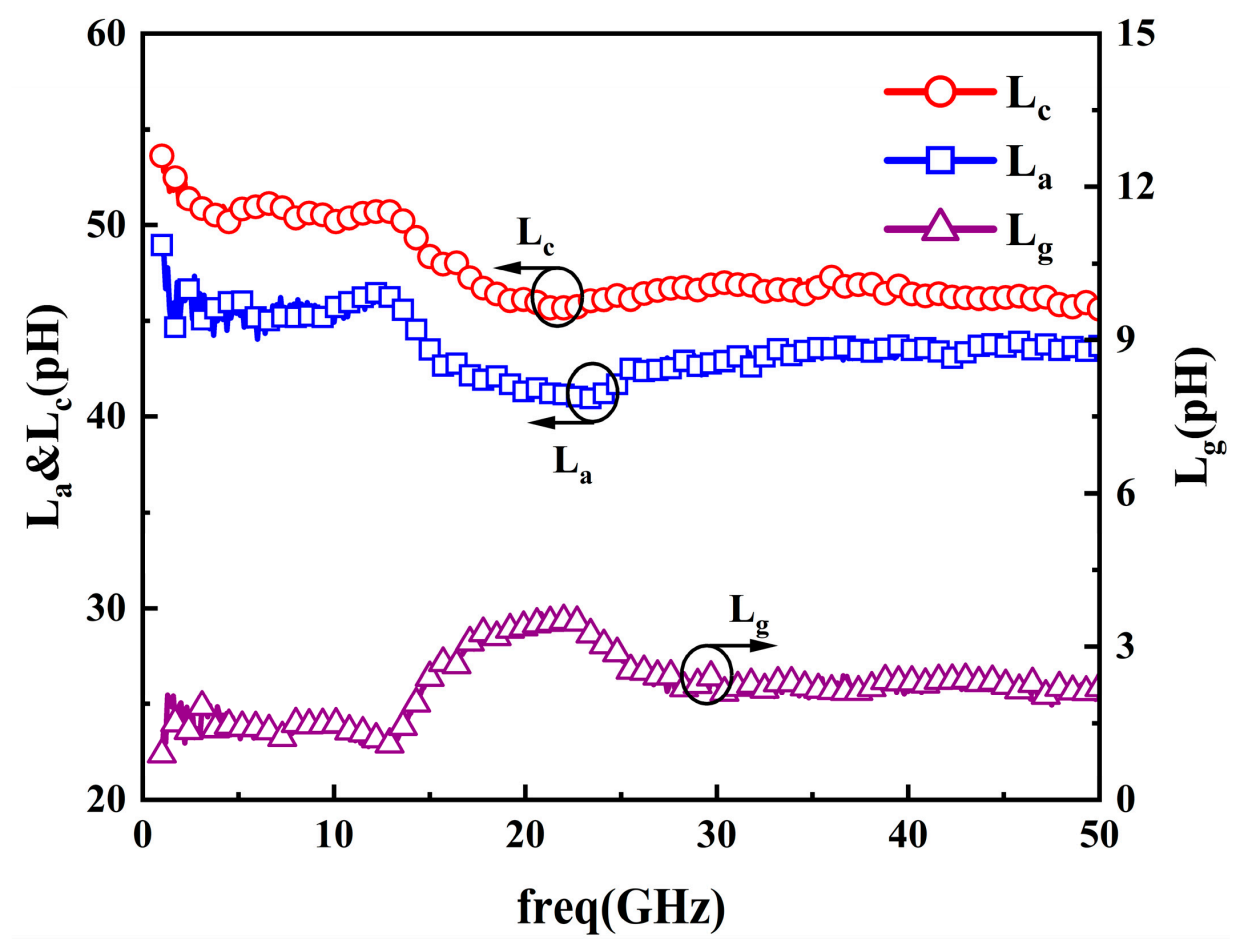
Parasitic inductances of the feedline.

**Figure 7:**
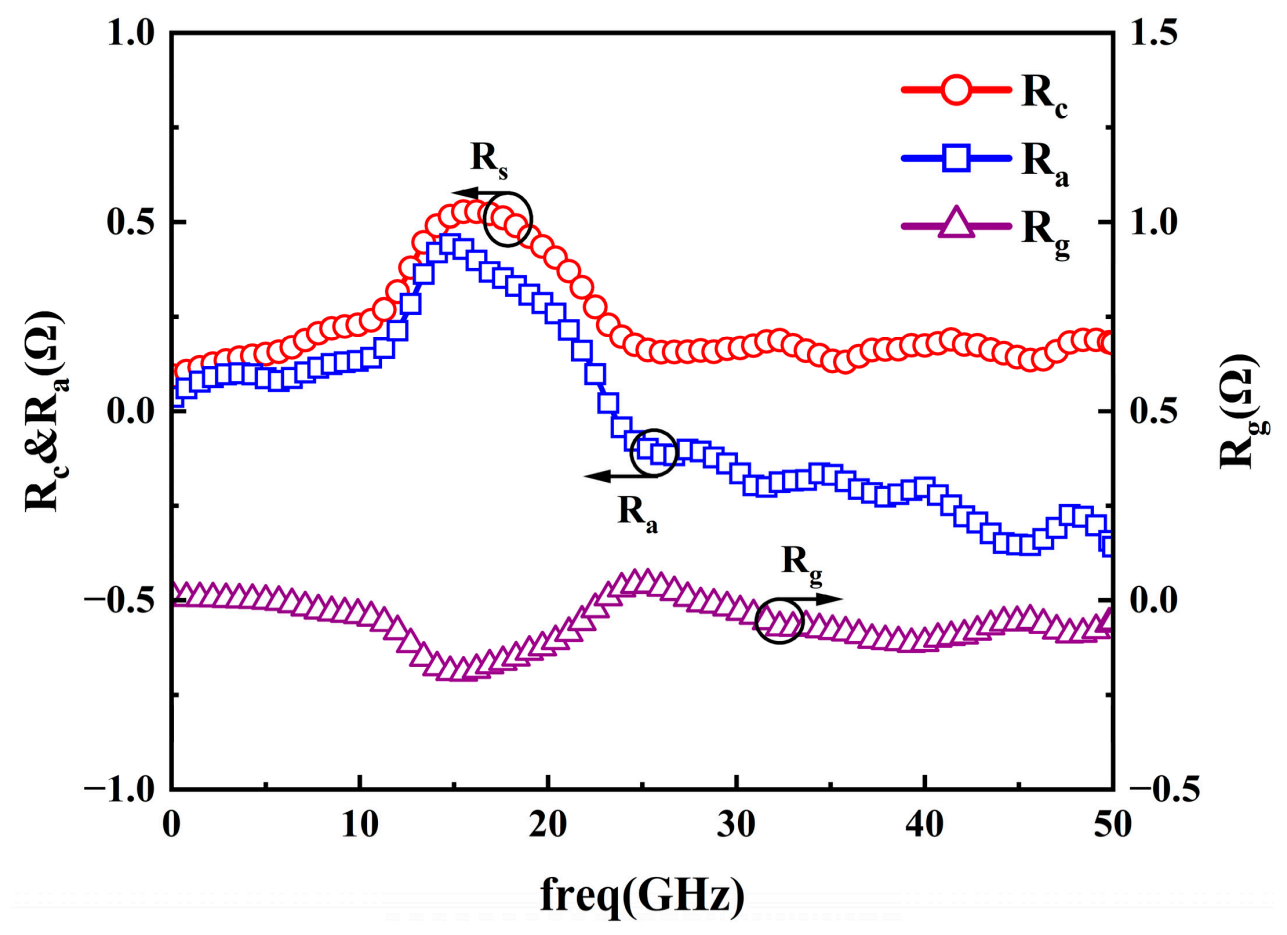
Parasitic resistances of the feedline.

Based on Equation (3), the expressions for the feedline parasitic parameters can be derived. Using the measured S-parameters as the basis, these parameters were likewise extracted in ADS, and the extraction results are presented in Figure 6 and Figure 7.

As can be seen from the extraction results in Figure 5, Figure 6 and Figure 7, the parasitic parameters exhibit small fluctuations and no noticeable frequency drift over the 0.1~50 GHz range, and their values are essentially frequency-independent. This confirms the reliability of the parasitic parameter extraction in ADS based on the measured GSG S-parameters, and provides a reliable foundation for the subsequent intrinsic small-signal modeling [12,14,15].

To ensure that the open and short models can accurately characterize the parasitic behavior in the high-frequency range up to 50 GHz, the measured S-parameters of the open and short test structures were used as the reference standard after the preliminary parasitic parameter extraction. The parameters were iteratively optimized in ADS to minimize the deviation between the simulated results and the measured data, enabling the optimized circuit models to more faithfully and accurately reflect the parasitic characteristics across the high-frequency test band. The optimized parameter values are listed in Table 1 below.

**Figure 8:**
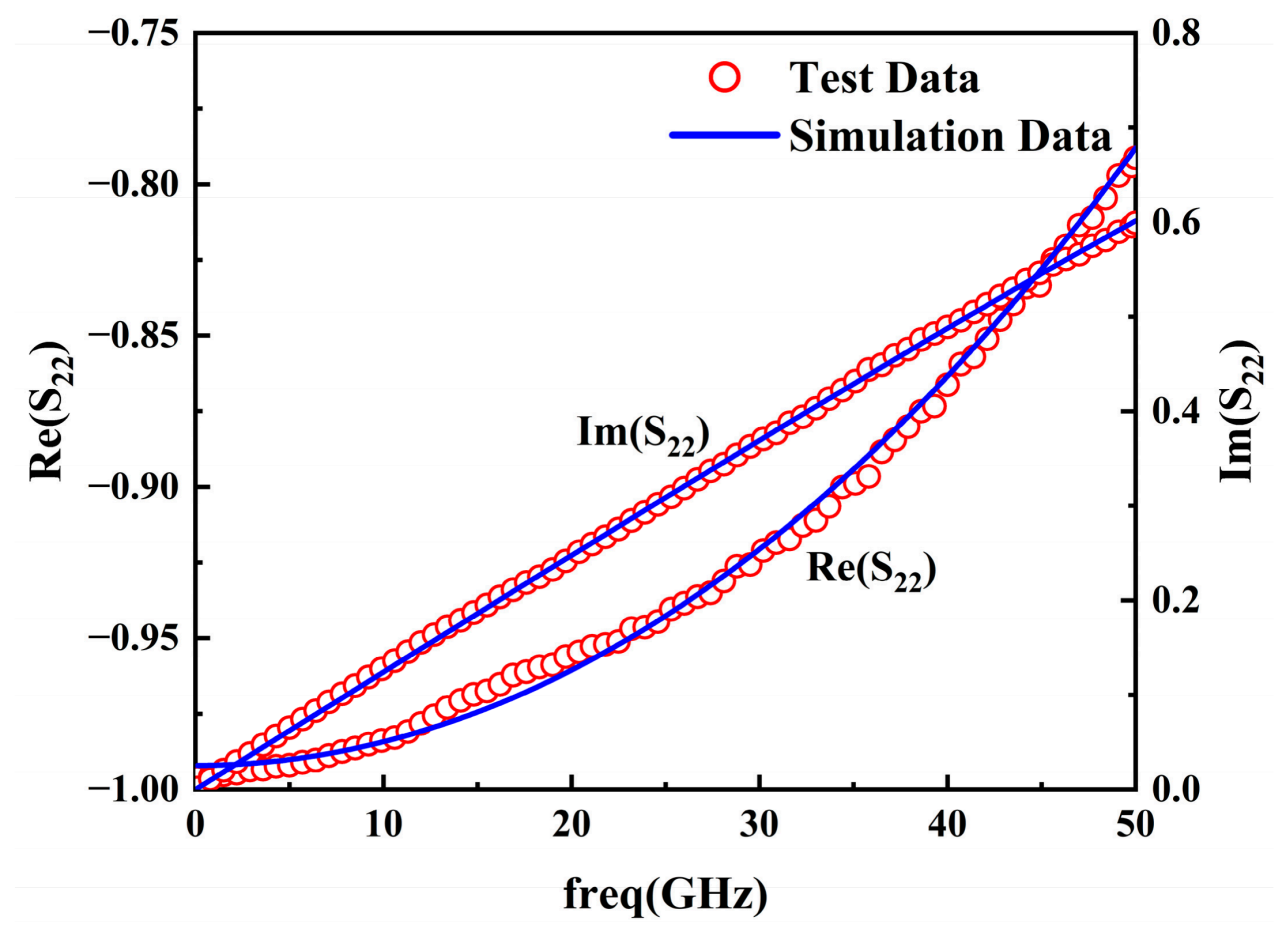
*S*_22_ parameter analysis of the short-circuit model.

Figure 8 shows a comparison between the measured and simulated *S*_22_ parameters of the short model. The discrete data points represent the measured S-parameters directly obtained from the vector network analyzer, while the curve represents the simulated results calculated from the short equivalent circuit model constructed in ADS. As can be seen from the figure, the measured and simulated *S*_22_ parameters are in excellent agreement over the frequency range of 0~50 GHz. To quantitatively evaluate the fitting performance, the root mean square error (RMSE) was calculated: the RMSE of the real part of *S*_22_ for this short structure is 0.0034, and that of the imaginary part is 0.0029. These results confirm that the parasitic circuit simulation is highly consistent with the measured data, indicating that reliable parasitic parameter de-embedding has been accomplished. Only by eliminating the interference introduced by the pads and feedlines can the true intrinsic RF parameters of the device be obtained, thereby providing a credible data foundation for the construction of the intrinsic small-signal equivalent circuit.

The extracted parasitic parameters show no obvious frequency dispersion across the 0.1–50 GHz band, which is consistent with the characteristics of GSG pad de-embedding reported in [14]. The fitting accuracy of the short model reaches the level required for conventional on-wafer microwave device characterization [3,7].

## 3. Small-Signal Modeling of GaN SBD

For semiconductor devices, the small-signal state means that when the device is biased at a certain DC operating point, the superimposed AC signal is sufficiently small, generally much smaller than the thermal voltage *kT*/*q*. Under such conditions, the electrical behavior of the device can be approximated as linear, which can be accurately described by a linear equivalent-circuit model [16,17,18,19].

### 3.1. De-Embedding

**Figure 9:**
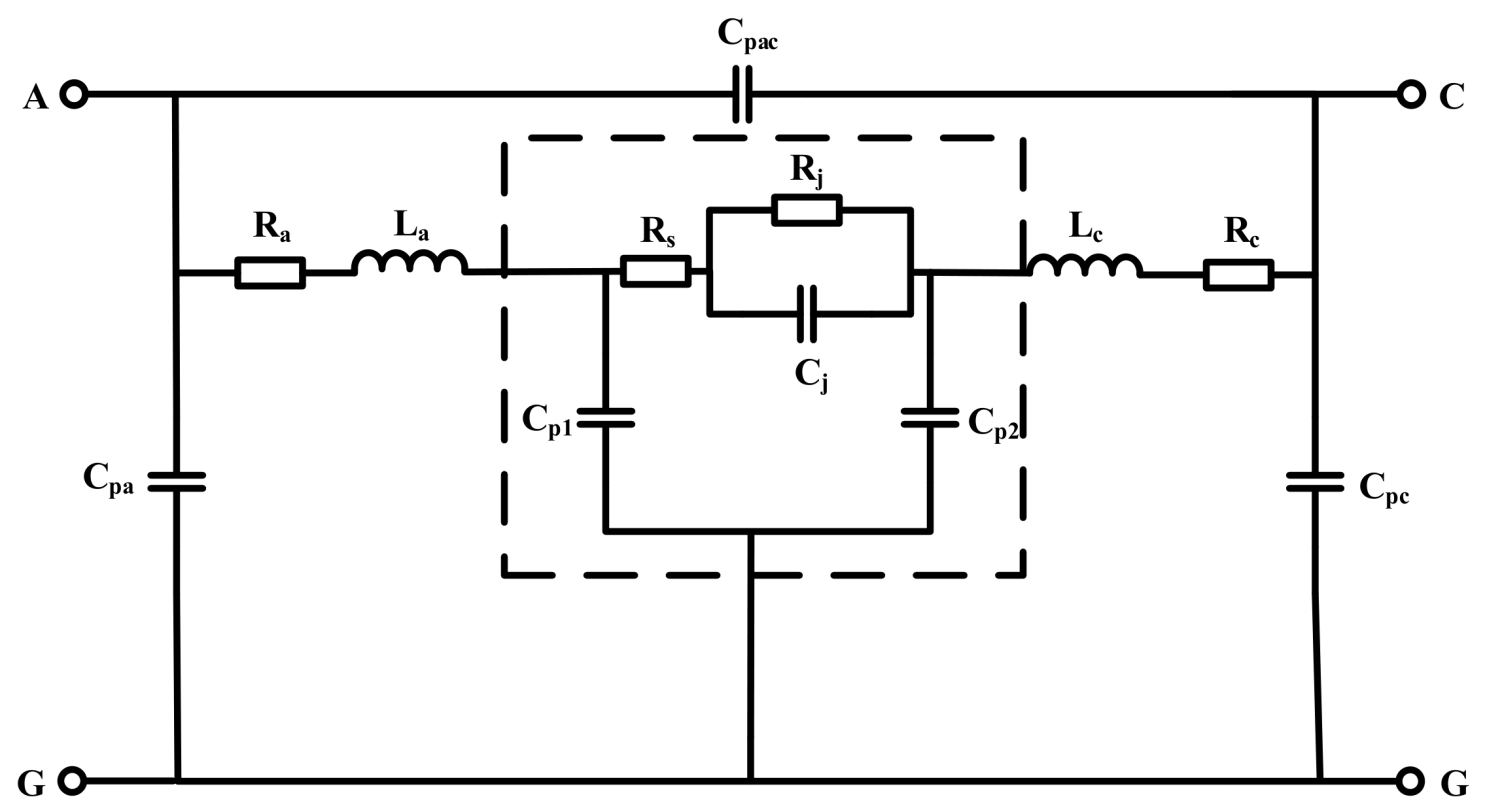
Small-signal model of the GaN SBD.

The complete small-signal model of the GaN SBD is presented below, as shown in Figure 9. The components enclosed within the dashed box represent the intrinsic parameters: *R_s_* is the pad resistance, *R_j_* and *C_j_* are the junction resistance and junction capacitance, respectively, and *C_p_*_1_ and *C_p_*_2_ are parasitic capacitances originating from the stray capacitances between internal device nodes and ground, which primarily arise from the coupling between layout interconnects and the dielectric layer. To extract these intrinsic parameters, the external parasitic parameters must first be de-embedded [16,17,18,19].

First, the S-parameters of the open and short test structures, *S_open_* and *S_short_*, and those of the device under test, *S_dut_*, are measured and then converted to Y-parameters: *Y_open_*, *Y_short_*, and *Y_dut_*.

Then, the external open parasitics are removed from the short structure [12,13,14,15,16]:

(4) $$Y_{short1}=Y_{short}-Y_{open}$$

It is converted to the Z-parameter matrix Z*_short_*_1_, and then the external open parasitics are removed from the DUT structure [12,13,14,15,16]:

(5) $$Y_{dut1}=Y_{dut}-Y_{open}$$

It is then converted to the Z-parameter matrix Zdut1 as well. Finally, the feedline parasitics in *Z_dut_*_1_ need to be removed. However, since the small-signal model of the GaN SBD does not include the ground feedline parasitics *R_s_* and *L_s_*, an additional Z-parameter matrix must be constructed [12,13,14,15,16]:

(6) $$Z_{short1}^{*}=\left[\begin{array}{cc} Z_{short1}(1,1)-Z_{short1}(1,2) & 0\\ 0 & Z_{short1}(2,2)-Z_{short1}(1,2) \end{array}\right]$$

where the *Z_short_*_1_ matrix is [12,13,14,15,16]:

(7) $$Z_{short1}=\left[\begin{array}{cc} R_{a}+R_{g}+j\omega (L_{a}+L_{g}) & R_{g}+j\omega L_{g}\\ R_{g}+j\omega L_{g} & R_{c}+R_{g}+j\omega (L_{c}+L_{g}) \end{array}\right]$$

Then $Z_{short1}^{*}$ is the Z-parameter matrix that does not contain the ground feedline parasitics. Finally, by removing the external feedline parasitics, the intrinsic Z-parameter matrix *Z_int_* of the GaN SBD can be obtained [12,13,14,15,16]:

(8) $$Z_{\mathrm{int}}=Z_{dut1}-Z_{short1}^{*}$$

Finally, the *Z_int_* matrix is converted to the Y-parameter matrix *Y_int_*, which represents the intrinsic Y-parameter matrix with the external parasitic parameters removed.

### 3.2. Intrinsic Parameter Extraction

Next, the pad resistance *R_s_* and the coupling capacitances *C_p_*_1_ and *C_p_*_2_ need to be extracted. For a Schottky diode, when *V_g_* = 3 V, the diode is under deep forward bias, and the junction resistance *R_j_* becomes extremely small and can be considered negligible. Moreover, since *C_p_*_1_ and *C_p_*_2_, as coupling capacitances, are insensitive to the bias voltage, the series resistance *Rs* and the coupling capacitances *C_p_*_1_ and C_p2_ are extracted at *V_g_* = 3 V.

**Figure 10:**
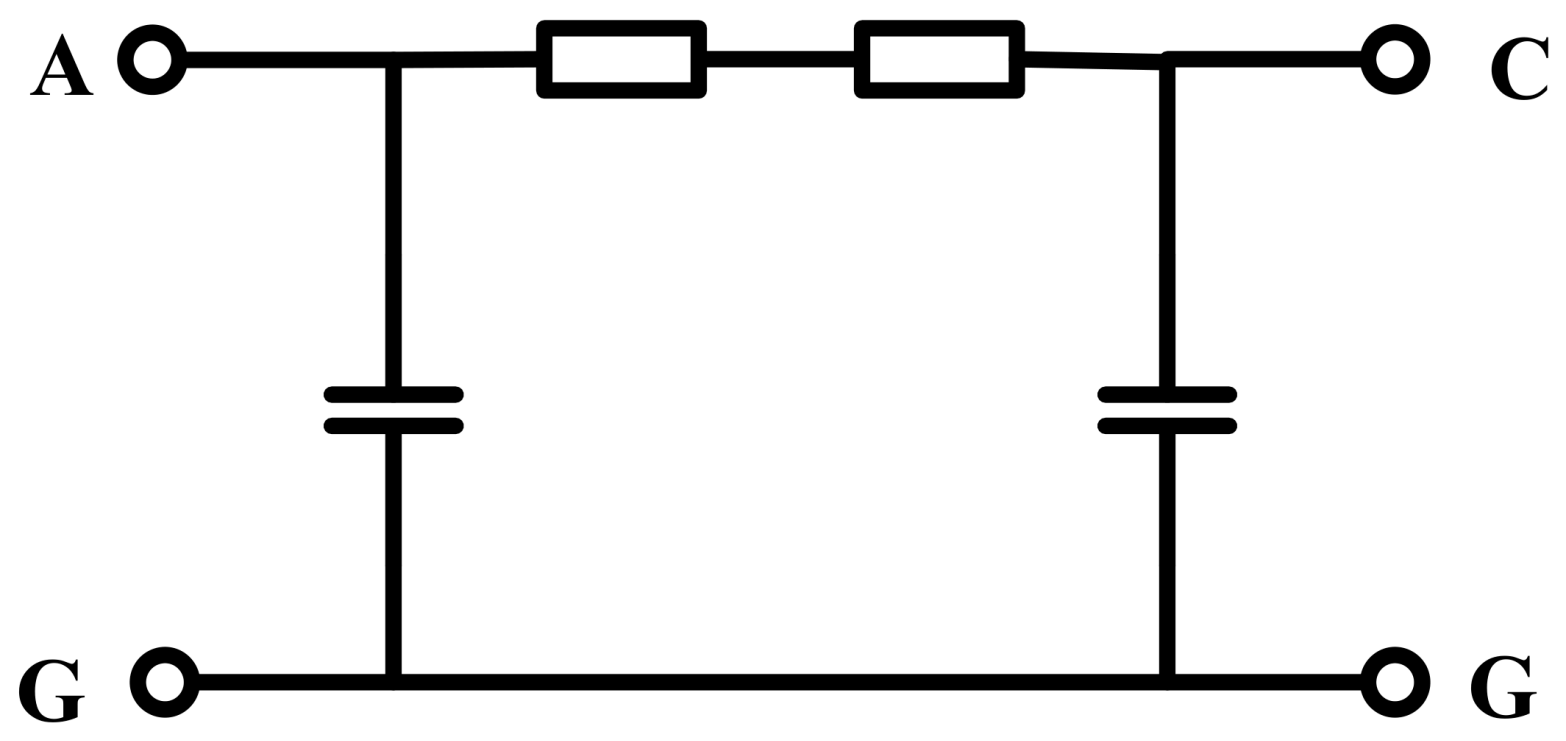
Small-signal model of the intrinsic part (Vg = 3 V).

At *V_g_* = 3 V, the small-signal model of the intrinsic part is shown in Figure 10:

From Figure 10, the intrinsic Y-parameter matrix Y_int_ can be obtained as follows [14,15,16]:

(9) $$Y_{\mathrm{int}}=\left[\begin{array}{cc} j\omega C_{p1}+\frac{1}{R_{s}+R_{j}} & -\frac{1}{R_{s}+R_{j}}\\ -\frac{1}{R_{s}+R_{j}} & j\omega C_{p2}+\frac{1}{R_{s}+R_{j}} \end{array}\right]$$

Based on the Y-parameter matrix, the expressions for the parasitic parameters are given in Equations (10)–(12) [14,15,16]:

(10) $$R_{s}=real(1/Y_{\mathrm{int}}(1,1))$$

(11) $$C_{p1}=\frac{imag(Y_{\mathrm{int}}(1,1)+Y_{\mathrm{int}}(1,2))}{\omega }$$

(12) $$C_{p2}=\frac{imag(Y_{\mathrm{int}}(2,2)+Y_{\mathrm{int}}(1,2))}{\omega }$$

**Figure 11:**
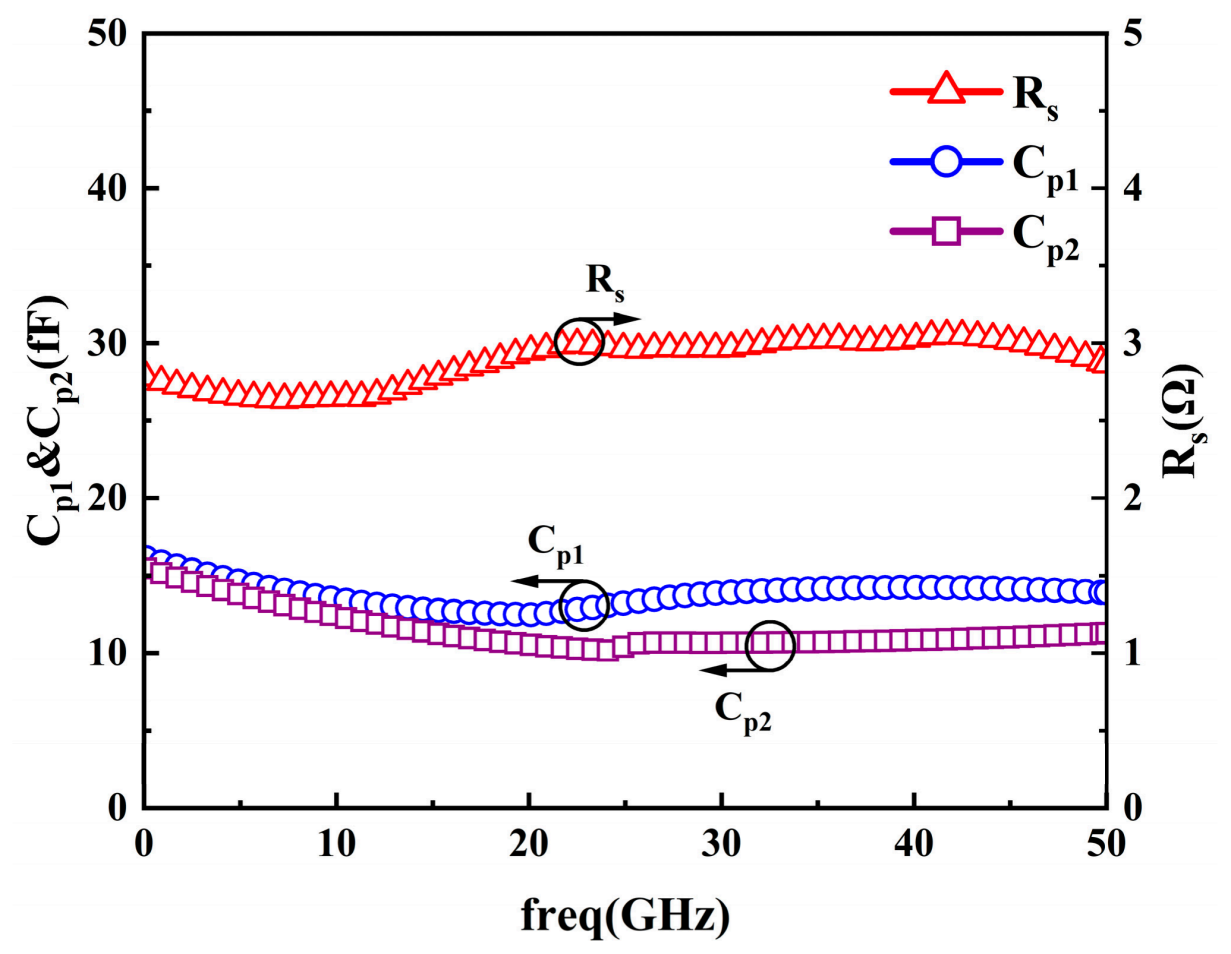
Frequency characteristics of *C_p_*_1_, *C_p_*_2_, and *R_s_*.

Based on Equations (10)–(12), the frequency-dependent characteristics of the parameters extracted in ADS using the measured S-parameters as the reference are shown in Figure 11.

The extracted parameters were optimized in ADS, and the resulting parameter values are listed in Table 2 below.

For a Schottky diode, the operating state of the junction varies significantly with bias voltage, and the dominant roles of the junction capacitance *C_j_* and junction resistance *R_j_* in the small-signal equivalent circuit differ accordingly. The bias range can be broadly divided into three regimes. Bias-dependent separation of capacitive and resistive junction behavior has also been used in equivalent-circuit descriptions of Schottky diodes [14,17,18].

1. Reverse bias to weak forward bias region (−8 V~0.2 V): This regime is dominated solely by the junction capacitance *C_j_*. Over this voltage range, the diode is either reverse-biased or in the cut-off state, the depletion region of the Schottky junction is relatively wide, and there is no significant forward injection of carriers. Consequently, the device behavior is governed primarily by the space-charge region capacitance effect, and using *C_j_* alone can accurately characterize the device in this regime.
2. Transition bias region (0.2 V~0.8 V): In this regime, *C_j_* and *R_j_* act jointly. This interval corresponds to the critical region where the diode transitions from cut-off to full conduction. The depletion region has not yet fully collapsed and still exhibits a capacitive effect, while forward carrier injection gradually intensifies and the junction conduction effect begins to emerge. Since capacitive and resistive effects coexist and are mutually coupled, a parallel configuration of *C_j_* and *R_j_* must be introduced to accurately capture the trend of the measured S-parameters and ensure the precision of the small-signal model in the transition region.
3. Forward bias region (0.8 V~3 V): This regime is dominated solely by the junction resistance *R_j_*. As the forward bias further increases, the depletion region width shrinks dramatically, leading to a significant rise in *C_j_*. Under these conditions, the value of *C_j_* becomes very large, and its capacitive impedance far exceeds the parallel equivalent impedance of *R_j_*. The shunting effect of the capacitance is severely suppressed, and its high-frequency contribution becomes negligible. Therefore, *R_j_* alone can accurately fit the small-signal characteristics in this regime.

**Figure 12:**
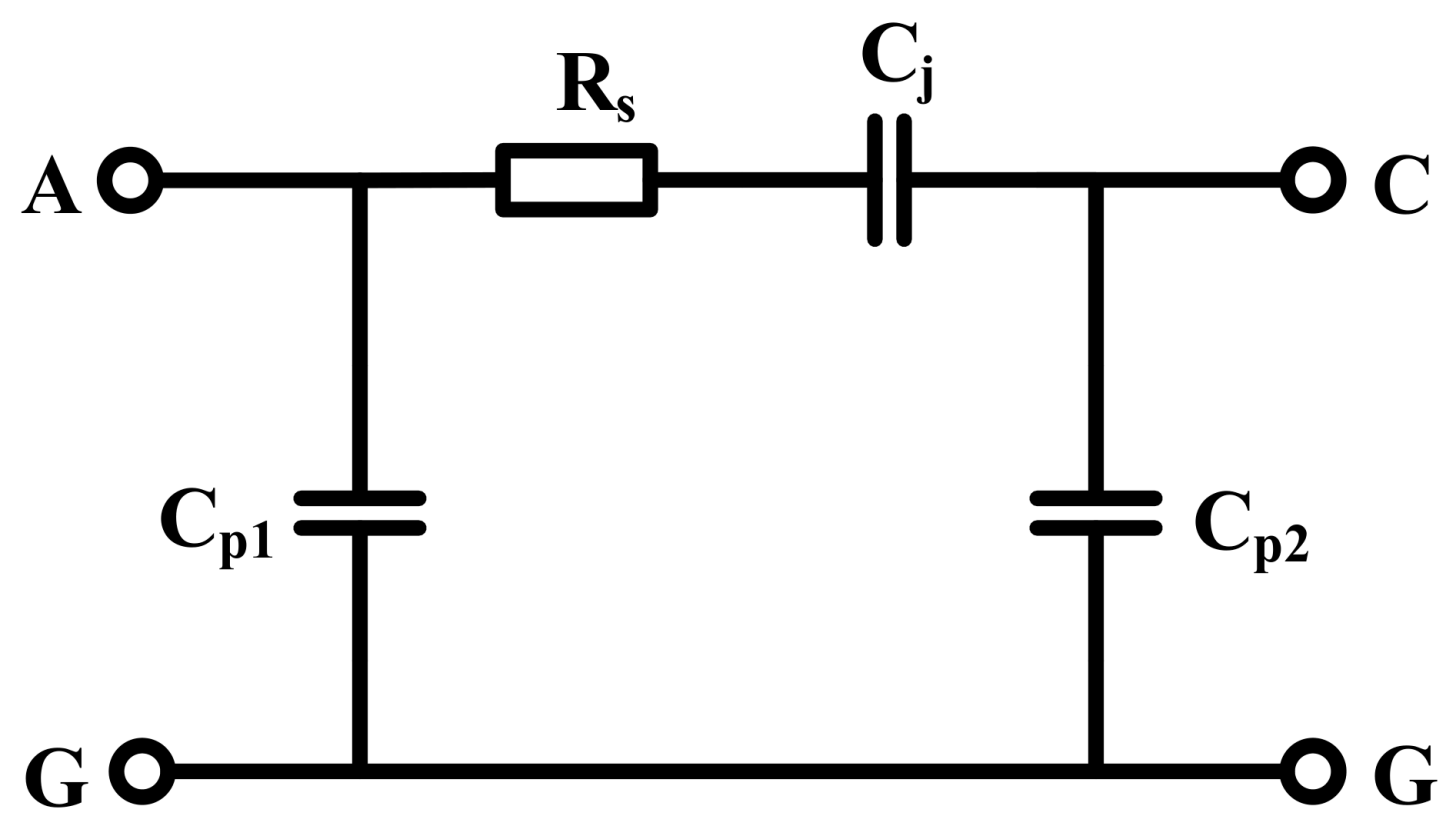
Small-signal model of the GaN SBD (Cj-dominated).

To characterize the bias-dependent behavior of the junction capacitance of the GaN SBD, a combined approach of measurement-based extraction and physical fitting is adopted in this work. Based on the de-embedded measured S-parameter data, the small-signal equivalent circuit model described earlier is employed, as shown in Figure 12.

Subsequently, the junction capacitance parameters at different bias voltages were obtained from the measured S-parameters. Parameter extraction and optimization were performed in ADS using the extraction formula given in Equation (13), and the resulting values are shown as the circular data points in Figure 12 [14,15,16].

(13) $$C_{j}=-\frac{1}{\omega \times imag(-\frac{1}{Y_{\mathrm{int}}(1,2)})}$$

**Figure 13:**
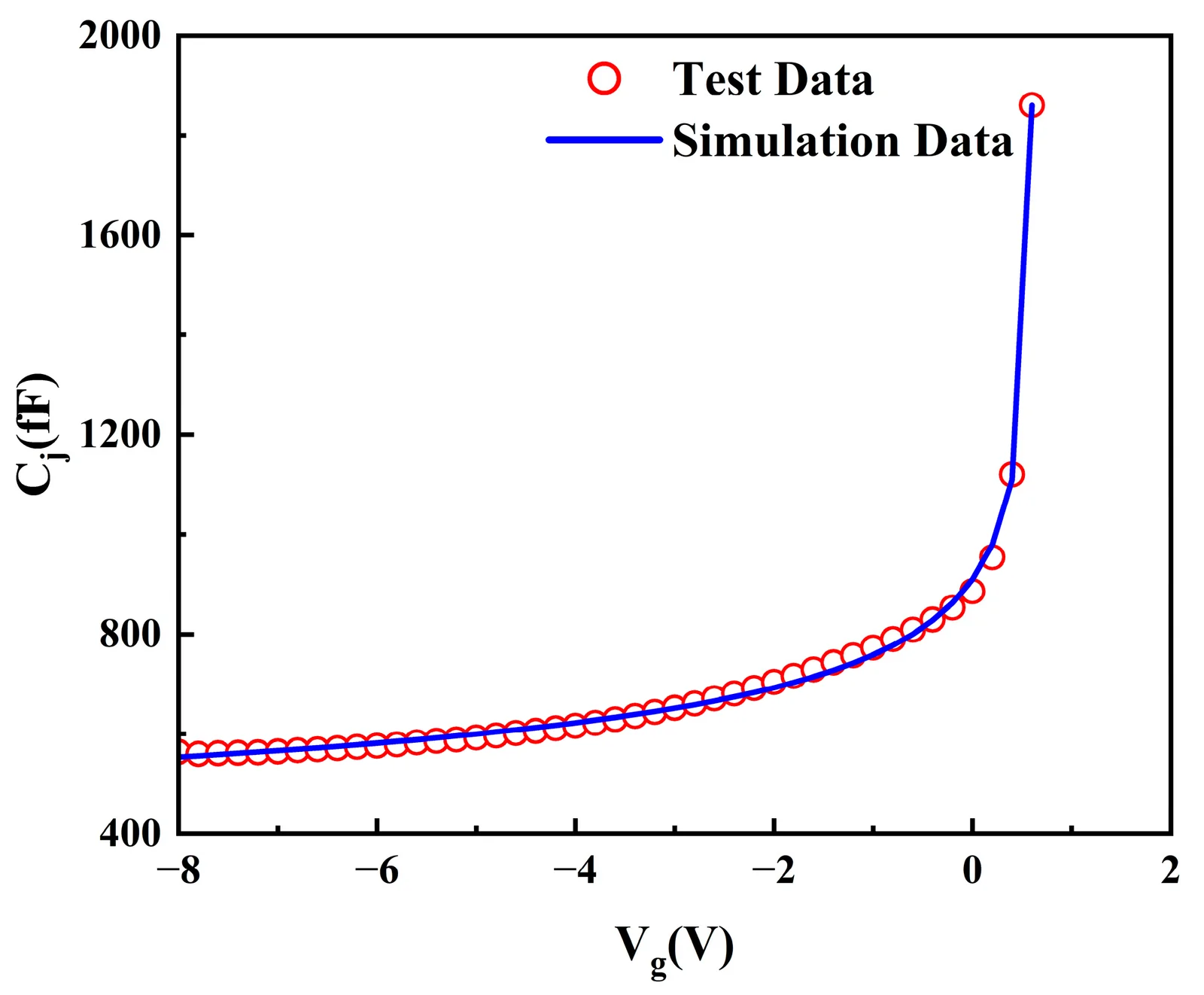
Junction capacitance Cj data curve.

Then, curve fitting was performed on the extracted *C_j_* data using the depletion capacitance model (Equation (14)), from which key physical parameters including the zero-bias junction capacitance *C_j_*_0_, built-in potential *V_bi_*, and grading coefficient *m* were extracted. The fitted curve is shown as the solid line in Figure 13, and the resulting RMSE = 9.2 fF, demonstrating that the depletion capacitance model can accurately match the extracted junction capacitance data [16,18].

(14) $$C_{j}=\frac{C_{j0}}{{\left(1-\frac{V_{g}-I_{d}Rs}{V_{bi}}\right)}^{m}}$$

The fitting results demonstrate that the parameter curve derived from the extraction and optimization of the measured S-parameters agrees well with the model-fitted curve over the bias range of −8 V~0.6 V, confirming the accuracy of the model parameters. The fitted parameter values are listed in Table 3.

**Figure 14:**
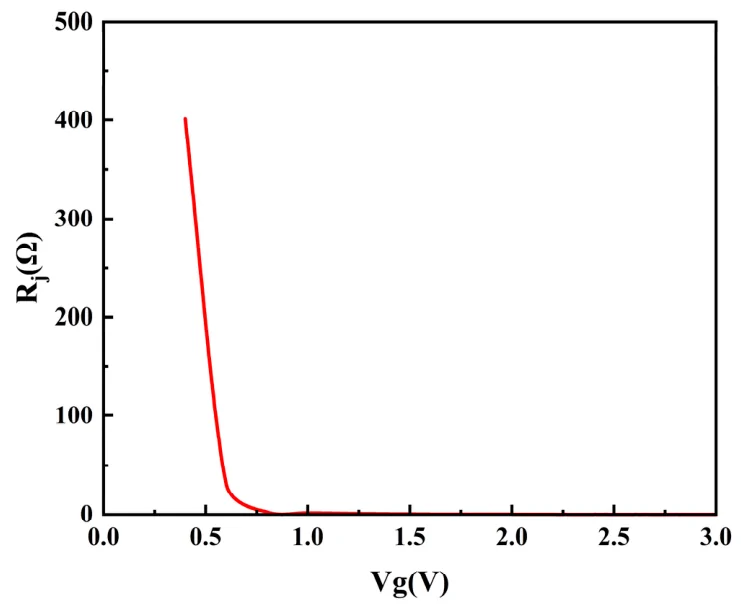
Junction resistance *R_j_* data curve.

Using the measured S-parameters at different bias voltages, the values of *R_j_* at various biases were extracted in ADS. Figure 14 presents the junction resistance *R_j_* as a function of voltage in the range of 0.4 V~3 V. The curve shows that the junction resistance decreases gradually with increasing bias voltage, exhibiting an overall trend of a rapid transition from a high-resistance state to a low-resistance state followed by stabilization. This behavior is highly consistent with the forward conduction physics of the Schottky diode.

**Figure 15:**
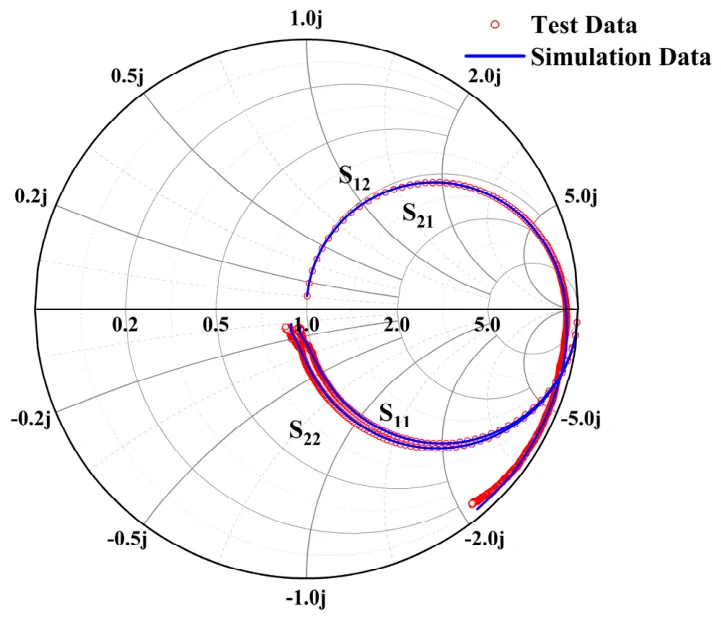
S-parameter comparison at Vg = −6 V.

**Figure 16:**
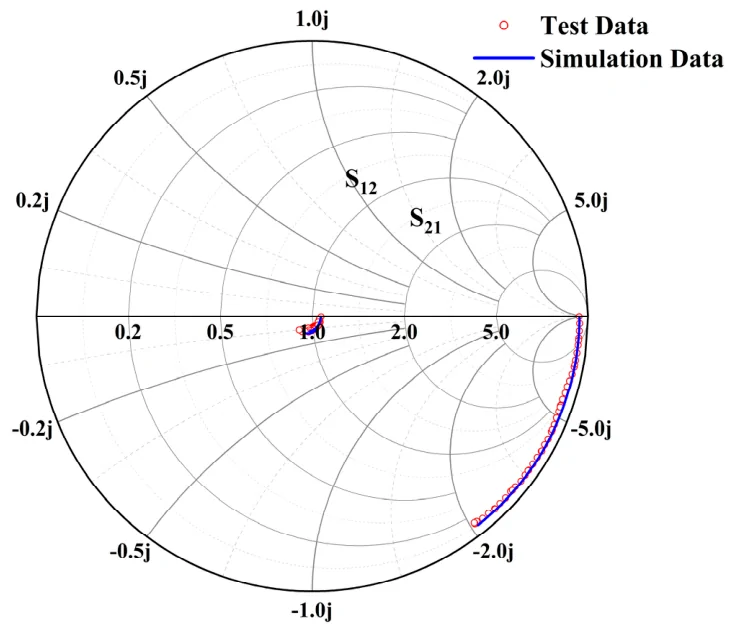
S-parameter comparison at Vg = 0 V.

**Figure 17:**
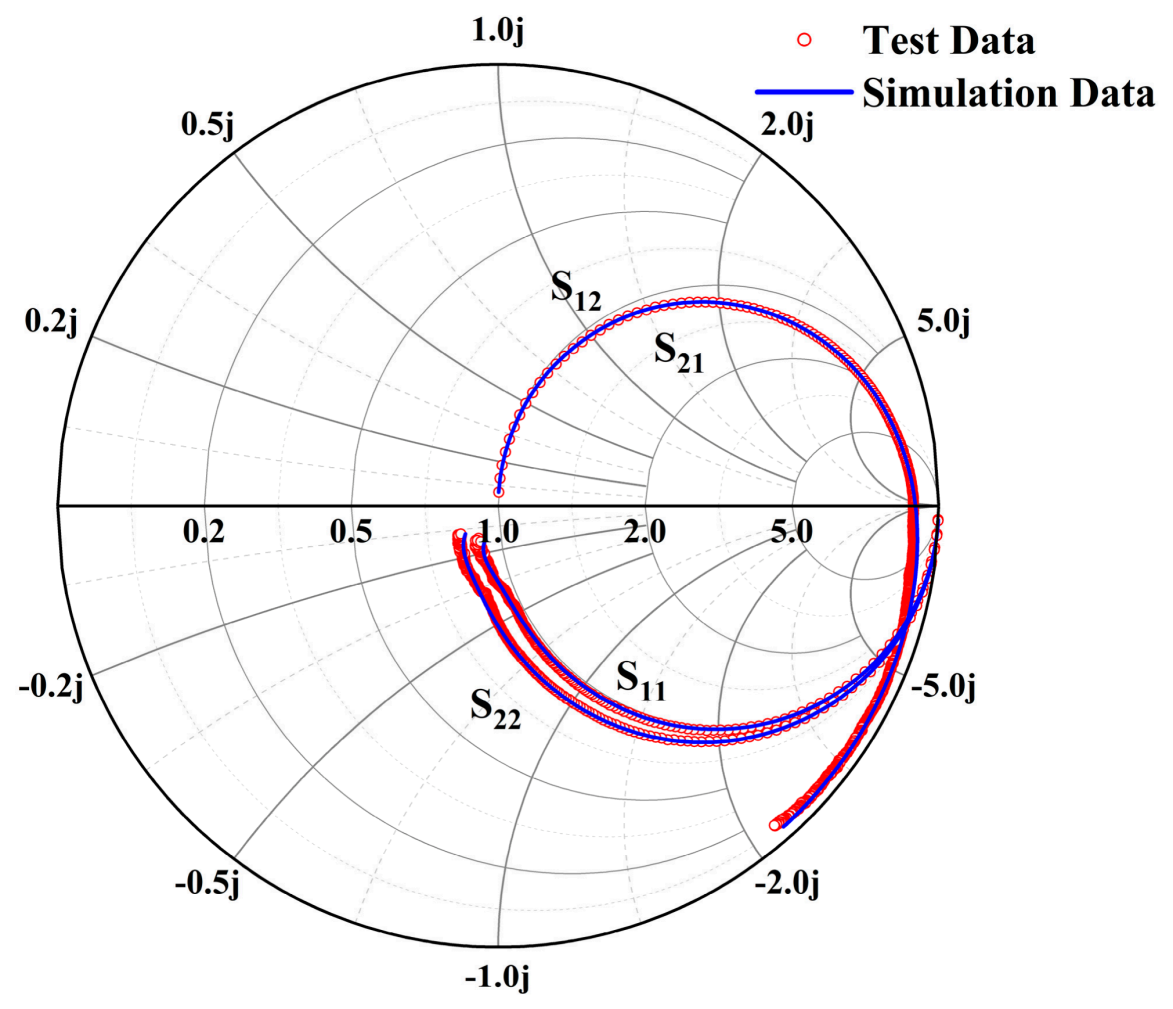
S-parameter comparison at Vg = 2 V.

Figure 15, Figure 16 and Figure 17 show Smith chart comparisons between the measured and simulated S-parameters of the device under three typical operating conditions: −6 V (reverse cut-off), 0 V (transition bias), and 2 V (deep forward conduction). The Smith charts intuitively reflect the complex impedance characteristics of the device as a function of frequency; the discrete symbols represent the measured S-parameters obtained directly from the vector network analyzer, and the solid lines denote the simulated S-parameter traces computed from the bias-dependent intrinsic small-signal equivalent circuit in ADS. The three bias points correspond to the three device operating regions defined in this work, and serve to verify the validity of the equivalent circuit topology under different operating states. As can be seen from the comparison figures, the measured data agree well with the simulated data. The specific RMSE values of the fitting are listed in Table 4 below. According to Table 4, all RMSE values are below 0.8, confirming the effectiveness and accuracy of the parameter extraction method proposed in this work [17,18,19].

The small-signal modeling described above has accomplished the RF characterization of the device. To fully describe the device’s operating behavior, it is also necessary to model and analyze the transport and leakage characteristics under DC bias.

## 4. DC Characteristic Analysis of GaN SBD

### 4.1. Forward DC Characteristics

This section further analyzes the forward DC characteristics of the device to characterize its intrinsic conduction performance. The circular data points in Figure 17 represent the measured forward current of the device as a function of voltage. The overall curve exhibits typical Schottky diode conduction behavior: the current increases exponentially with voltage in the low-bias region, and enters a linear conduction region at high bias, where it is limited by the series resistance.

**Figure 18:**
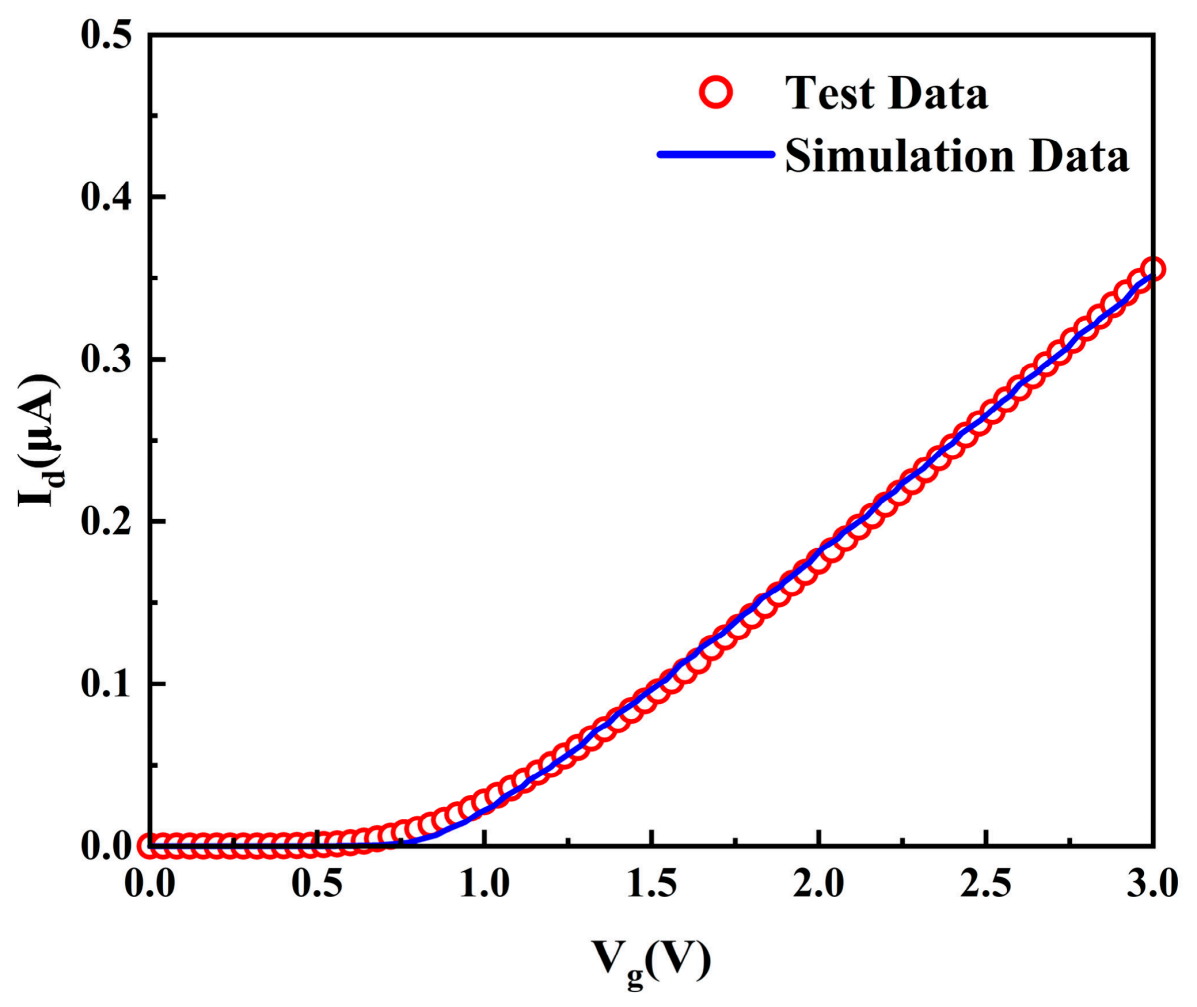
Forward I–V characteristic curve.

To extract the intrinsic transport parameters of the device, the classic Schottky thermionic emission model is adopted in this work to fit the measured forward I–V curve, and the model is given by Equation (15) [20,21].

(15) $$I_{d}=I_{s}\left(e^{\frac{q(V_{g}-I_{d}R_{s,dc})}{nkT}}-1\right)$$

where *I_s_* is the reverse saturation current, *n* is the ideality factor, *R_s,dc_* is the DC series resistance, *q* is the electron charge, *k* is the Boltzmann constant, and T is the absolute temperature. The fitted curve is shown as the solid line in Figure 18. The coefficient of determination *R*^2^ of the model fitting exceeds 0.999. As can be seen from the figure, the measured data exhibit excellent agreement with the fitted data over the voltage range of 0 V~3 V, indicating that the forward transport mechanism of the device is dominated by thermionic emission.

The fitted values of the intrinsic transport parameters are listed in Table 5 below.

To verify the self-consistency of the extracted intrinsic transport parameters, a differential analysis of the forward I–V curve was performed.

**Figure 19:**
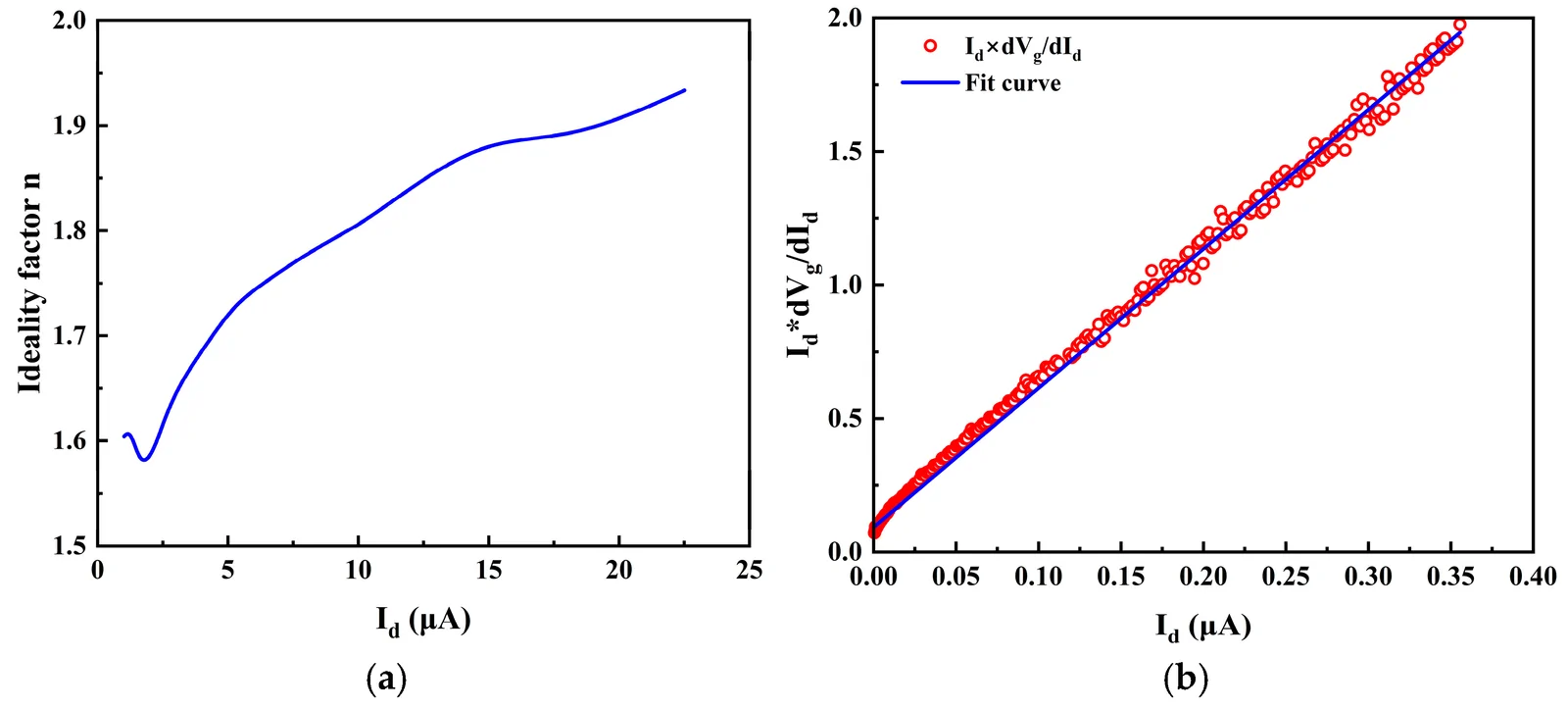
(**a**) Variation in the ideality factor *n* with forward current in the low-bias range. (**b**) Series resistance extraction plot: Id*dVg/*dId* versus Id.

Figure 19a shows the variation in the ideality factor *n* with current, calculated as follows:

(16) $$n=\frac{q}{kT}\frac{dV_{g}}{d(\ln I_{d})}$$

In the low-bias range of 0.15–0.3 V, *n* increases gradually from about 1.6 to nearly 1.9, with no obvious plateau. This trend originates from the spatial inhomogeneity of the Schottky barrier height: low-barrier regions conduct preferentially, and as the bias increases, high-barrier regions gradually participate in conduction, causing the apparent *n* to rise continuously. The overall magnitude is consistent with the global fitting value of 1.84. Figure 19b shows the *I_d_* × *dV_g_*/*dI_d_* versus *I_d_* curve in the range of 0.5–3 V. The curve exhibits good linearity, and the series resistance extracted from the fitting slope is approximately 5.2 Ω. This value is slightly lower than the 5.6 Ω obtained from the global fitting; the deviation arises from different fitting constraints: the global fitting simultaneously optimizes three parameters, whereas the differential method extracts the resistance only from the linear region. The two values are of the same order of magnitude, verifying the reliability of the parameters.

The DC series resistance *R_s,dc_* = 5.6 Ω, obtained from fitting the DC measurement data, and the series resistance *R_s,rf_* = 3.3 Ω previously extracted from high-frequency S-parameter measurements exhibit a significant discrepancy. This discrepancy cannot be simply attributed to measurement or extraction errors; rather, it reflects an important physical effect in GaN devices—the trap-state-induced frequency dispersion effect. Under DC measurement conditions, the bias voltage varies slowly, allowing deep-level traps (such as carbon-related acceptor defects or nitrogen-vacancy complexes) located in the GaN epitaxial layer and at the metal–semiconductor interface sufficient time to respond. These traps capture or emit carriers, establishing occupation states that follow the changing bias conditions; the resulting change in net charge density modulates the width of the space-charge region and the degree of band bending, thereby increasing the effective series resistance of the device and leading to a higher *R_s,dc_*. In contrast, under high-frequency small-signal excitation (0.1~50 GHz), the signal period is much shorter than the trap emission/capture time constants, and the trap occupation states cannot follow the rapidly alternating bias conditions to charge and discharge. Consequently, the trap charge density is approximately “frozen” at an average occupation state corresponding to the DC bias point, greatly reducing its modulation of the space-charge region. The device thus exhibits an intrinsic series resistance unaffected by dynamic trap modulation, resulting in a smaller *R_s,rf_*. This phenomenon is a typical dispersion effect caused by material defect states in wide-bandgap semiconductors [22].

Furthermore, the ideality factor *n* = 1.84 obtained from the forward I-V characteristic fitting is significantly higher than the ideal value of unity, indicating the presence of non-ideal factors in the forward transport. This deviation is mainly attributed to the spatial inhomogeneity of the Schottky barrier height (Barrier Height Inhomogeneity, BHI). Factors such as interface states, dislocations, and surface roughness cause the local Schottky barrier height to exhibit a statistical distribution, and the total current is a superposition of multiple parallel conduction paths with different barrier heights, resulting in an apparent ideality factor greater than unity. Despite these non-ideal factors, thermionic emission (TE) remains the dominant conduction mechanism within the forward bias range tested in this work. The complete TE equation incorporating the series resistance correction term (Equation (15)) achieves high-precision fitting *R*^2^ > 0.999 to the measured data over the full range of 0–3 V, demonstrating that the current–voltage relationship follows the functional form of the TE model. Therefore, *n* = 1.84 should be understood as the manifestation of the TE model under spatially inhomogeneous barrier conditions, rather than a failure of the TE mechanism [23,24,25].

### 4.2. Reverse DC Characteristics

The reverse leakage current characteristic of GaN SBDs is a critical indicator for evaluating their breakdown performance and reliability. The dotted part in Figure 19 shows the device’s leakage current characteristic curve under a reverse bias from −100 V to 0 V. It can be observed that as the reverse bias increases, the leakage current exhibits an overall trend of initially increasing and then saturating.

The reverse leakage current of GaN SBDs is governed by multiple physical mechanisms, among which common ones include thermionic emission (TE), PF emission, Schottky barrier lowering (SBL), trap-assisted tunneling (TAT), Fowler–Nordheim (FN) tunneling, and variable-range hopping (VRH). Among these possible leakage mechanisms, TE under reverse bias typically yields a nearly constant, voltage-independent saturation current at a given temperature, which cannot account for the observed reverse I-V characteristics. FN tunneling requires extremely high electric fields (generally > MV/cm), which are not reached under the measurement conditions in this study. VRH dominates primarily at cryogenic temperatures or in heavily disordered materials and is therefore unlikely to play a significant role at room temperature. Consequently, this work focuses on three mechanisms: SBL, TAT, and PF emission [24,25,26,27,28].

The SBL effect originates from the image force. When an electron is emitted from the metal into the semiconductor, an equal amount of positive charge is induced on the metal surface. The resulting image charge exerts an attractive force on the electron, reducing its potential energy at the interface. This is equivalent to superimposing a negative image potential onto the original Schottky barrier. Taking the image force into account, the potential energy of an electron near the interface can be expressed as [20,24,27]:

(17) $$E(x)=-\frac{q^{2}}{16\pi \varepsilon_{s}x}-qE_{m}x$$

where *q* is the electron charge, *ε_s_* is the semiconductor permittivity, and *E_m_* is the maximum electric field at the metal–semiconductor interface. The position of the barrier maximum *x_m_* and the barrier lowering Δϕ can be obtained by setting *dE*/*dx* = 0, yielding [20,24,27]:

(18) $$\Delta \phi =\sqrt{\frac{qE_{m}}{4\pi \varepsilon_{s}}}$$

For a uniformly doped Schottky abrupt junction, the maximum electric field Em is related to the reverse bias voltage *V_g_* by [20,24,27]:

(19) $$E_{m}=\sqrt{\frac{2qN_{d}}{\varepsilon_{s}}\left(V_{bi}+V_{g}-\frac{kT}{q}\right)}$$

where *N_d_* is the doping concentration and *V_bi_* is the built-in potential. When the reverse bias is sufficiently large (*V_g_* ≫ *V_bi_*), Em is approximately proportional to $V_{g}^{1/2}$, and substituting this into the expression for Δϕ yields $\Delta \phi \propto V_{g}^{1/4}$.

Based on the thermionic emission model, substituting the effective barrier height $\phi_{B}=\phi_{B0}-\Delta \phi$ into the current equation yields the reverse SBL current as [20,24,27]:

(20) $$I_{SBL}=I_{0}e^{\frac{q\Delta \phi }{kT}}$$

Its simplified form can be written as [20,24,27]:

(21) $$I_{SBL}=I_{0}e^{\gamma V_{g}^{1/4}}$$

where *I*_0_ is the zero-bias equivalent saturation current, which is directly related to the barrier height $\phi_{B0}$, and γ is a constant associated with the doping concentration and the dielectric constant of the material.

TAT is a process in which electrons traverse the Schottky barrier using defect energy levels as “stepping stones.” Compared with direct tunneling, TAT has a higher tunneling probability and constitutes a significant leakage path in GaN materials with high defect densities. At a constant temperature, the TAT current can be expressed as [24,25,26,27]:

(22) $$I_{TAT}\propto e^{-\frac{8\pi \sqrt{2m^{*}}}{3qhE}\phi_{t}^{3/2}}$$

where m* is the electron effective mass, *h* is Planck’s constant, $\phi_{t}$ is the depth of the trap energy level relative to the conduction band edge, and E is the electric field in the depletion region. For an abrupt junction, the electric field E varies with voltage approximately as $E\propto V^{1/2}$; therefore, the dominant term of the TAT current takes the form $\exp(\alpha V^{-1/2})$. However, in practical fitting, to simplify parameters and focus on the overall current growth trend, the TAT model is often further simplified as [24,25,26,27]:

(23) $$I_{TAT}=I_{T0}e^{\alpha V}$$

where *I_T_*_0_ is the equivalent saturation current, and α is a coefficient that comprehensively reflects the trap depth and tunneling probability.

**Figure 20:**
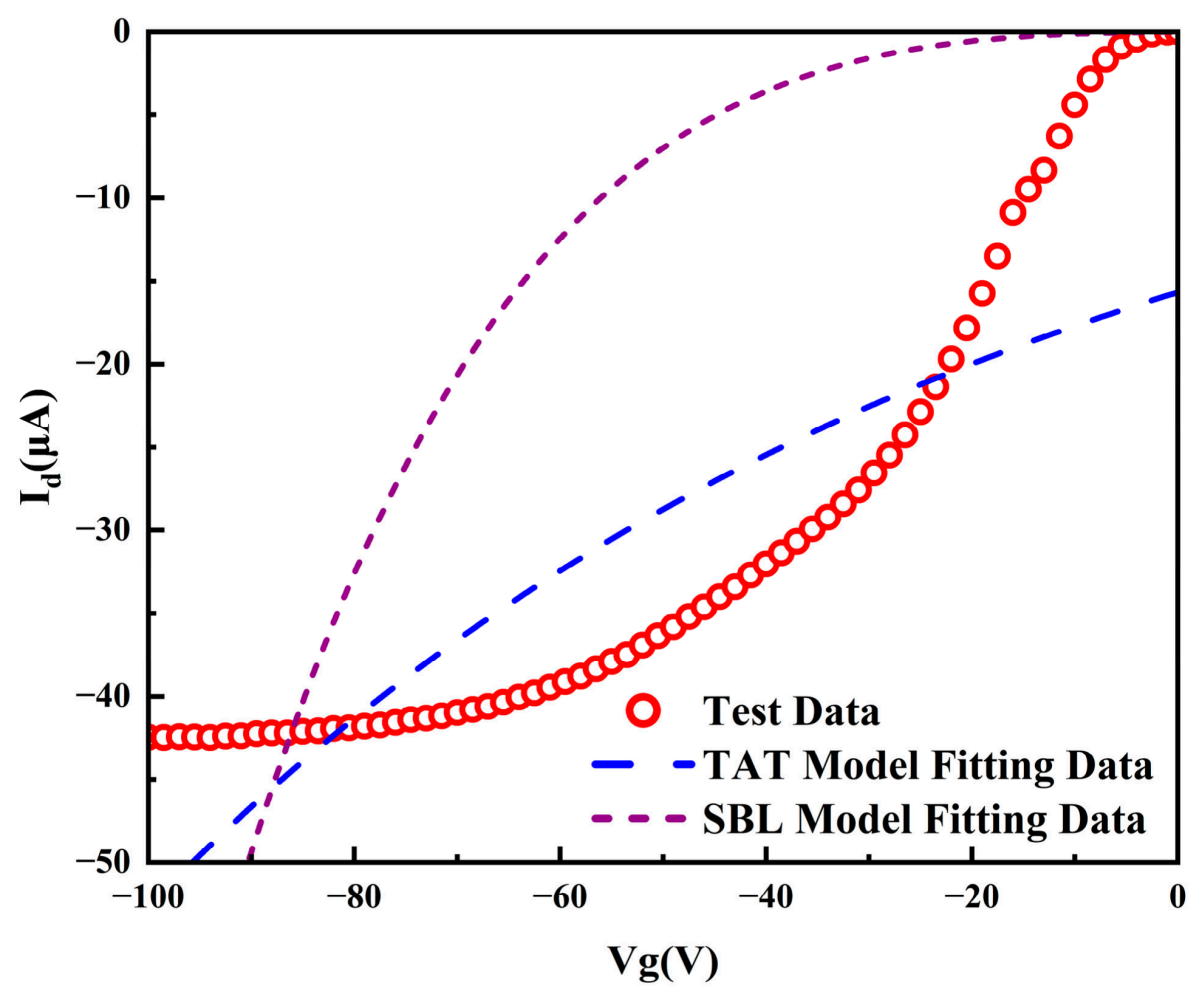
Fitted curves of the SBL and TAT models.

The reverse I–V characteristic curve was fitted using Equations (21) and (23), and the fitting results are shown in Figure 20. The fitting results indicate that neither the SBL mechanism, which is dominated by interface effects, nor the TAT mechanism, which is dominated by bulk defects, can independently describe the reverse leakage behavior of the device.

Based on the above analysis, a piecewise modeling strategy is adopted in this work. In the low reverse bias region of −14 V~0 V, the leakage current is dominated by the PF trap-assisted emission mechanism. The classical expression for the PF current is [24,26,27]:

(24) $$I\propto E\cdot e^{-\frac{q\phi_{t}}{kT}}\cdot e^{\frac{q}{kT}\sqrt{\frac{q^{3}E}{\pi \varepsilon_{s}}}}$$

**Figure 21:**
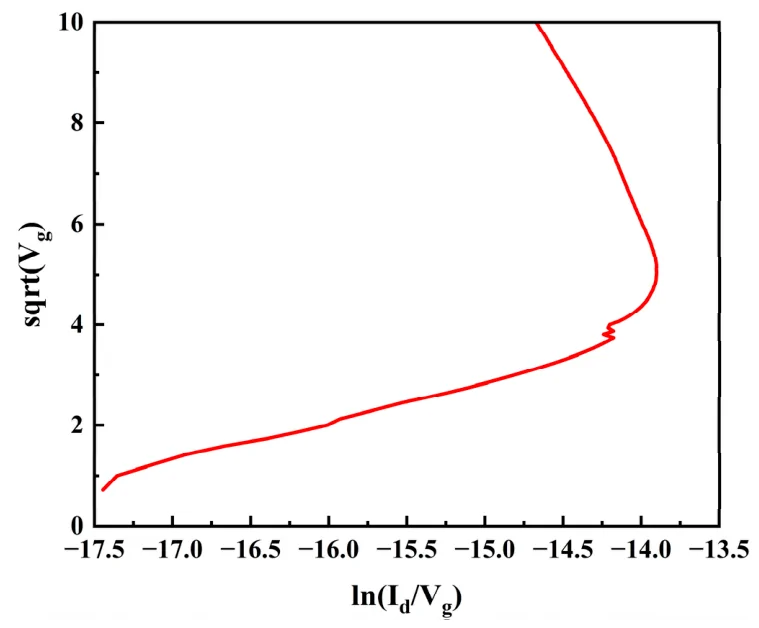
Curve of *ln*(*I_d_*/*V_g_*) as a function of $V_{g}^{1/2}$.

For a Schottky depletion layer, under high reverse bias conditions, the electric field approximately satisfies $E\propto V^{1/2}$. Substituting this relationship into the above equation and absorbing constants such as temperature and material permittivity into fitting parameters yields the simplified form of the PF model [24,26,27]:

(25) $$I_{d}=AV_{g}e^{\frac{\beta \sqrt{V_{g}}}{kT}}$$

where *A* is a constant, *β* is the PF coefficient, and *V_g_* is the reverse bias voltage. According to the theoretical characteristics of the PF model, if the leakage current is dominated by the PF emission mechanism, then *ln*(*I_d_*/*V_g_*)exhibits a clear linear relationship with $V_{g}^{1/2}$. Figure 21 shows the curve of *ln*(*I_d_*/*V_g_*) as a function of $V_{g}^{1/2}$. From the figure, it can be seen that in the low reverse bias range of −14 V~0 V, the curve exhibits good linearity, indicating that the leakage current in this voltage range is primarily governed by the PF trap-assisted emission mechanism, with the current dominated by carrier emission assisted by defect levels in the GaN material. However, as the reverse bias further increases (Below −14 V), the curve no longer conforms to the linear relationship of the PF model, indicating that the dominant leakage mechanism has changed and the PF trap-assisted emission model is no longer applicable.

**Figure 22:**
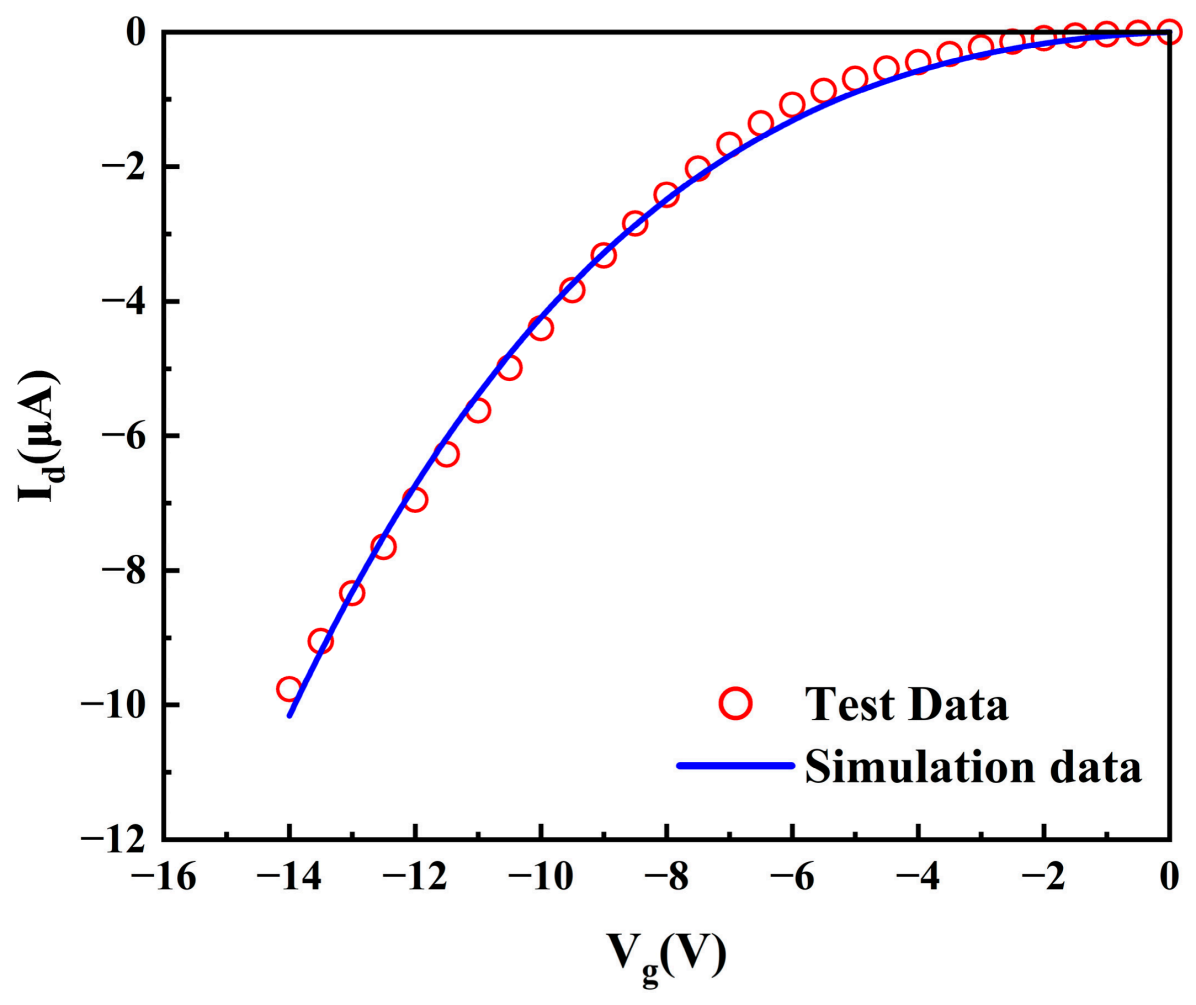
Reverse I-V characteristic curve in the range of −14 V~0 V.

Figure 22 shows a comparison between the measured leakage current data and the PF model fitting curve in the reverse bias range of −14 V~0 V. As can be seen from the figure, the measured data and the fitted data are in good agreement in the reverse voltage range of 014 V~0 V; *R*^2^ > 0.997, indicating that the leakage current in the low reverse bias region is primarily governed by the trap-assisted emission mechanism [24,26,27].

The fitting results are as follows: the constant *A* = 9.61 × 10^−9^ and the PF emission coefficient *β* = 0.031. *β* reflects the extent to which the defect energy level is reduced by the electric field. This result indicates that in the low reverse bias range of –14 V~0 V, the defect energy levels within the device are significantly lowered by the applied electric field, and defect-assisted carrier emission becomes the dominant leakage transport process [24,26,27].

**Figure 23:**
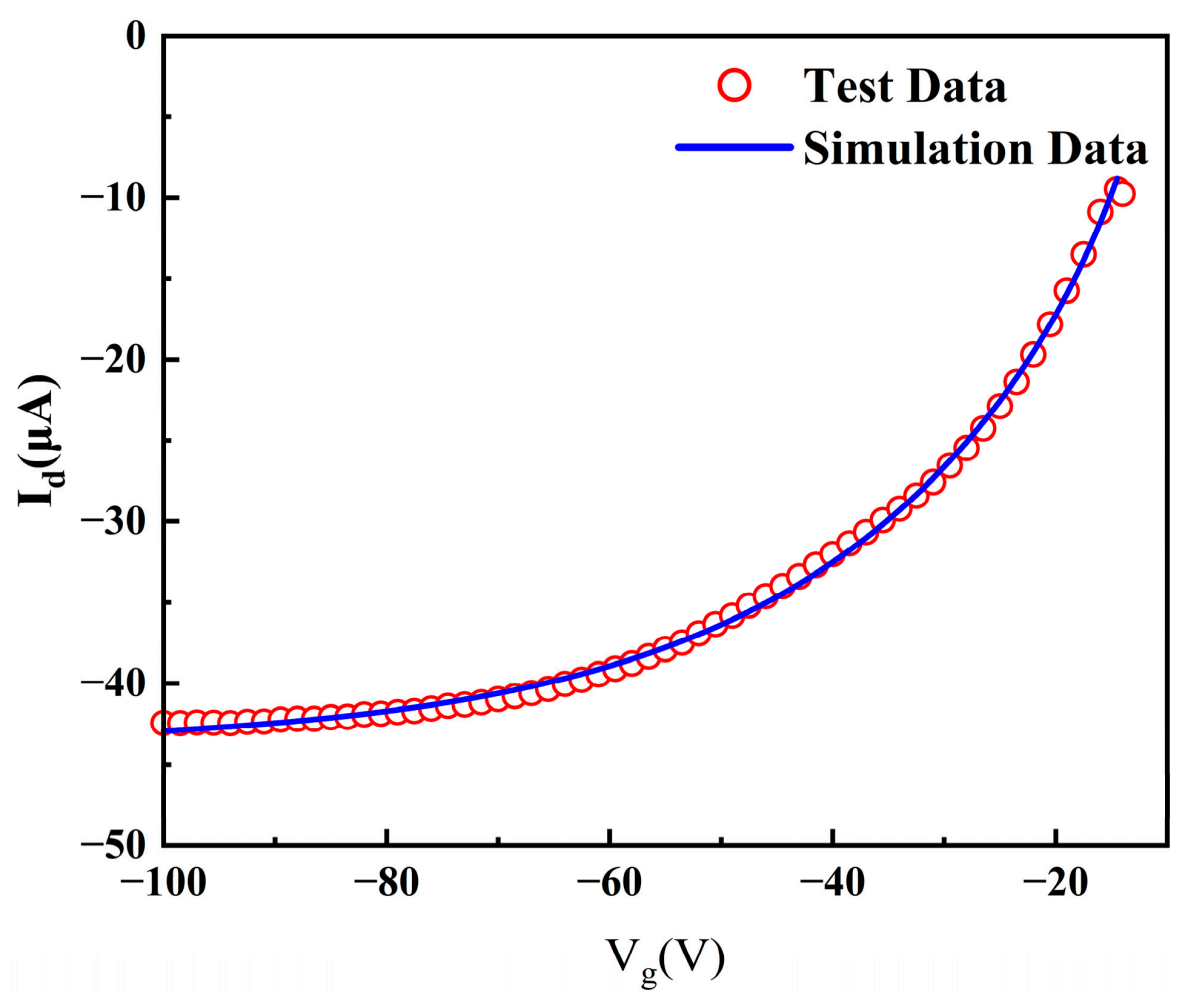
Reverse I-V characteristic curve in the range of −100 V~−14 V.

When the reverse bias further increases above −14 V, the rate of current increase with voltage slows down markedly, and the current gradually approaches a saturation value, indicating that the single PF emission mechanism applicable at low voltages is no longer valid. To quantitatively characterize this saturation-like growth behavior of the leakage current in the −100 V~−14 V range, this paper proposes an empirical model in the form of a double-exponential decay. Empirical modeling has been proven effective for describing complex transport behavior in Schottky diodes [29]; however, the specific double-exponential form adopted here is proposed for the GaN SBD in this work and is expressed as:

(26) $$I_{d}=I_{sat}-A_{1}e^{\left(-\frac{V_{g}}{\tau_{1}}\right)}-A_{2}e^{\left(-\frac{V_{g}}{\tau_{2}}\right)}$$

where *I_sat_* is the saturation current at high bias, *A*_1_ and *A*_2_ are amplitude coefficients, and *τ*_1_ and *τ*_2_ are characteristic voltages that describe the rate of increase in the leakage current. This double-exponential model is an empirical fitting formula, and its parameters do not possess strictly analytical physical meanings: *τ*_1_ = 4.14 V corresponds to the rapid increase stage of the leakage current at low bias, and *τ*_2_ = 24.1 V corresponds to the gradual saturation stage of the current at high bias. The comparable magnitudes of *A*_1_ and *A*_2_ indicate that the contributions of both stages to the total leakage current are non-negligible. The saturation of the leakage current at high bias arises from the coupling of multiple mechanisms and is difficult to describe with a single analytical formula. This empirical model can effectively fit the evolution of the leakage current over the range of −100 V to −14 V, and the fitting results are shown in Figure 23.

As can be seen from the figure, the fitted results of this empirical formula are in excellent agreement with the measured data in the reverse voltage range of −100 V~−14 V, with a goodness of fit R^2^ > 0.998, indicating a very high fitting accuracy. The fitted parameter values are listed in Table 6.

**Figure 24:**
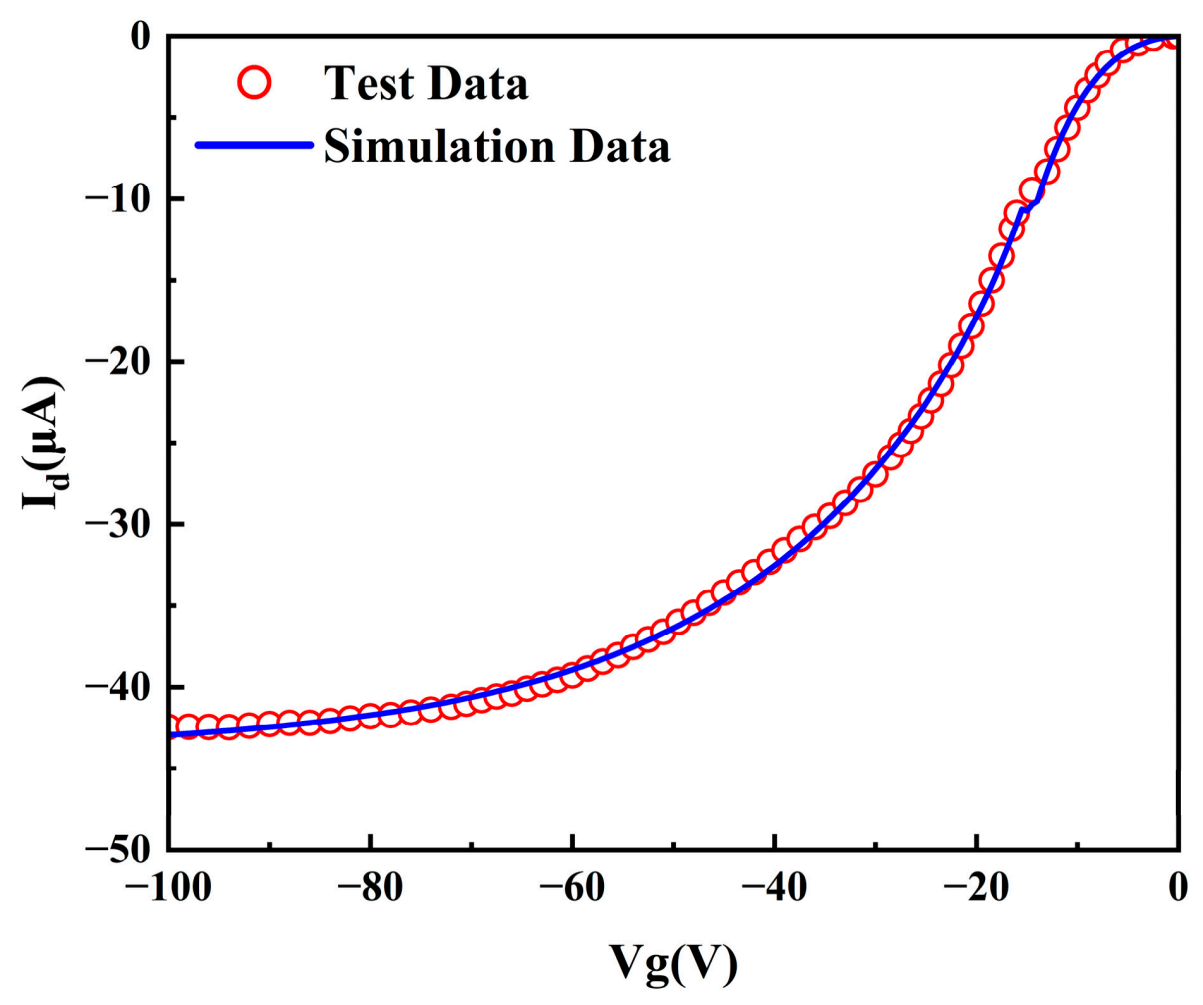
Reverse I-V characteristic curve in the range of−100 V~0 V.

Figure 24 presents the fitted I-V characteristic curve under reverse bias from −100 V to 0 V. In the −14 V~0 V range, the PF model accurately describes the leakage behavior dominated by trap-assisted emission; in the −100 V~−14 V range, the double-exponential decay model precisely captures the evolution characteristics of the current gradually approaching saturation. The two curves transition smoothly at the junction point, and the goodness of fit *R*^2^ exceeds 0.998 over the full voltage range. Furthermore, the overall current fitting RMSE = 2.85 × 10^−7^ A, and the piecewise model exhibits excellent agreement over the full bias range, indicating that the adopted piecewise modeling strategy can completely and accurately describe the leakage characteristics of the GaN SBD over the entire reverse bias range [27,29].

Compared with the fitting effect of a single physical mechanism model, the piecewise strategy achieves higher fitting accuracy over the full reverse bias range, which is consistent with the conclusion of composite leakage model studies in [11,24].

## 5. Discussion

This paper establishes a comprehensive GaN Schottky diode modeling framework covering parasitic stripping, bias-dependent small-signal modeling, and full-range DC characterization, with an in-depth mechanism analysis of all measured and fitted results. At the high-frequency parasitic treatment level, the open–short de-embedding method can independently separate the pad parasitic capacitance, feedline inductance, and ohmic series resistance. The extracted parameters exhibit negligible frequency drift over 0.1–50 GHz, and the extremely small RMSE of the short model directly demonstrates that this method effectively eliminates parasitic interference, thereby ensuring the extraction accuracy of the intrinsic device parameters. This behavior is consistent with previously reported Schottky-diode de-embedding and parameter-extraction approaches [3,7,8,12,13,14,15,16], while the present work verifies the parasitic and intrinsic models within a unified 0.1~50 GHz and DC modeling flow.

After parasitic de-embedding, the RF behavior of the device is dominated by the competing relationship between the junction capacitance *C_j_* and junction resistance *R_j_*. Consequently, this paper divides the device into three regions—reverse cut-off, transition, and deep forward conduction—and matches the corresponding equivalent topologies. This partitioning approach conforms to the carrier transport physics under different biases. The extracted junction capacitance variation fully agrees with depletion capacitance theory. The S-parameters at three typical biases of −6 V, 0 V, and 2 V all exhibit extremely low fitting errors, confirming that the bias-dependent modeling can fully reproduce the high-frequency response over the entire 0.1~50 GHz bandwidth. The bias-dependent equivalent-circuit treatment and the observed capacitance/resistance evolution are also consistent with prior RF and planar Schottky-diode modeling studies [17,18,19].

The forward DC transport is primarily governed by the thermionic emission mechanism, with the corresponding I–V curve fitting achieving a goodness-of-fit *R*^2^ > 0.999, indicating high agreement between the model and measurement. The extracted ideality factor of *n* = 1.84 is significantly higher than the ideal value of unity. This deviation is primarily attributed to the spatial inhomogeneity of the Schottky barrier height at the metal/GaN interface, where factors such as interface states, dislocations, and surface roughness result in an apparent ideality factor greater than unity. Furthermore, a significant discrepancy exists between the series resistance extracted from DC measurements (*R_s,dc_* = 5.6 Ω) and that extracted from RF measurements (*R_s,rf_* = 3.3Ω). This discrepancy originates from the different response behaviors of deep-level traps under DC and RF excitation—namely, the trap-induced frequency dispersion effect. In contrast, the reverse leakage current exhibits two distinct evolution behaviors: at low bias, trap-assisted PF emission dominates, while at high bias the current gradually saturates. A single physical formula cannot simultaneously cover both processes, directly causing large fitting deviations. To address this, this paper adopts a piecewise strategy, employing the PF model in the low-voltage region and introducing a double-exponential empirical model in the high-voltage region. The fitting accuracy is uniformly quantified using *R*^2^ and RMSE. The overall current RMSE over the full reverse bias range is only 2.85 × 10^−7^ A, numerically confirming the significant accuracy advantage of the piecewise approach over a single-transport model. The thermionic-emission-dominated forward transport and the defect-associated reverse leakage behavior are in agreement with reported Schottky and GaN current-transport studies [23,24,25,26,27,28]. Compared with mechanism-focused single-regime descriptions, the present reverse model combines the low-bias PF mechanism with a high-bias empirical saturation description [29] in one full-range fitting framework.

Integrating the fitting results of the parasitic, RF, and DC aspects, all simulated curves exhibit excellent consistency with the measured data. The entire integrated modeling workflow resolves the insufficient fitting accuracy inherent in traditional single-model, non-partitioned methods. The extracted high-frequency model is also relevant to the modeling requirements reported for GaN/planar Schottky devices in terahertz and frequency-conversion applications [30,31,32].

## 6. Conclusions

This paper establishes an integrated parasitic de-embedding, bias-dependent small-signal, and DC modeling framework for GaN Schottky diodes. High-precision parasitic parameter extraction over the 0.1~50 GHz frequency range is realized using an open–short method. Based on the competing relationship between the junction capacitance and junction resistance, a three-region small-signal equivalent circuit is constructed to accurately reproduce the device’s high-frequency characteristics. The forward DC transport is dominated by the thermionic emission mechanism. The extracted ideality factor of *n* = 1.84 is attributed to the spatial inhomogeneity of the Schottky barrier height at the metal/GaN interface. Furthermore, a significant discrepancy exists between the series resistance extracted from DC measurements (5.6 Ω) and that from RF measurements (3.3 Ω), which is attributed to the trap-induced frequency dispersion effect. The core innovation lies in the proposed piecewise fitting strategy for the reverse leakage current: a PF trap emission model is adopted in the low-voltage region, and a double-exponential empirical model is introduced in the high-voltage region to describe the current saturation effect. Over the full bias range of −100 V to 0 V, the current fitting RMSE is only 2.85 × 10^−7^ A, resolving the large fitting deviation of a single transport model between low and high biases. The overall forward *R*^2^ exceeds 0.999, and the S-parameter errors are extremely low across the entire frequency band. The simulation results are in excellent agreement with measurements, providing a complete modeling solution for RF circuit and reliability simulations of similar devices. Future work can extend to wide-temperature and large-signal nonlinear modeling to further refine the framework.

## Figures and Tables

**Table 1:** Optimized values of the parasitic parameters.

Parameter	Value	Unit	Parameter	Value	Unit
*C_pac_*	1.7	fF	*L_c_*	16.5	pH
*C_pa_*	19.16	fF	*R_c_*	194.8	mΩ
*C_pc_*	20.43	fF	*L_g_*	2.28	pH
*L_a_*	43.25	pH	*R_g_*	1.1	mΩ
*R_a_*	1.06	mΩ			

**Table 2:** Optimized values of the intrinsic parasitic parameters.

Parameter	Value	Unit
*R_s_*	3.3	Ω
*C_p_*_1_	12	fF
*C_p_*_2_	10	fF

**Table 3:** Fitted parameters of the junction capacitance model.

Parameter	Value	Unit
*C_j_*_0_	909	fF
*V_bi_*	0.614	V
*m*	0.188	-

**Table 4:** RMSE values between the measured and fitted data at biases of −6 V, 0 V and 2 V.

	Vg = −6 V	Vg = 0 V	Vg = 2 V
RMSE_S_11_	0.304 dB	0.513 dB	0.612 dB
RMSE_S_12_	0.058 dB	0.073 dB	0.068 dB
RMSE_S_21_	0.057 dB	0.073 dB	0.069 dB
RMSE_S_22_	0.278 dB	0.582 dB	0.708 dB

**Table 5:** Values of the intrinsic transport parameters.

Parameter	Value	Unit
*I_s_*	7.37 × 10^−10^	A
*R_s,dc_*	5.6 Ohm	Ω
*n*	1.84	-

**Table 6:** Fitted parameter values of the empirical model for the bias range of−100 V~−14 V.

Parameter	Value	Unit
*I_sat_*	4.39 × 10^−5^	A
*A*_1_	7.82 × 10^−5^	-
$\tau_{1}$	4.14	V
*A*_2_	5.97 × 10^−5^	-
$\tau_{2}$	24.1	V

## Data Availability

The simulation and test data used to support the findings of this study are included within the figure files and can be obtained by contacting the authors (aozhang@ntu.edu.cn and chengjl@jou.edu.cn).
